# O-GlcNAcylation of YTHDF2 promotes HBV-related hepatocellular carcinoma progression in an N^6^-methyladenosine-dependent manner

**DOI:** 10.1038/s41392-023-01316-8

**Published:** 2023-02-10

**Authors:** Yang Yang, Yu Yan, Jiaxin Yin, Ni Tang, Kai Wang, Luyi Huang, Jie Hu, Zhongqi Feng, Qingzhu Gao, Ailong Huang

**Affiliations:** Institute for Viral Hepatitis, Key Laboratory of Molecular Biology for Infectious Diseases (Ministry of Education), Department of Infectious Diseases, The Second Affiliated Hospital, Chongqing Medical University, Chongqing, China

**Keywords:** Tumour virus infections, Gastrointestinal cancer, Oncogenesis

## Abstract

Hepatitis B virus (HBV) infection is a major risk factor for hepatocellular carcinoma (HCC), but its pathogenic mechanism remains to be explored. The RNA N^6^-methyladenosine (m^6^A) reader, YTH (YT521-B homology) domain 2 (YTHDF2), plays a critical role in the HCC progression. However, the function and regulatory mechanisms of YTHDF2 in HBV-related HCC remain largely elusive. Here, we discovered that YTHDF2 O-GlcNAcylation was markedly increased upon HBV infection. O-GlcNAc transferase (OGT)-mediated O-GlcNAcylation of YTHDF2 on serine 263 enhanced its protein stability and oncogenic activity by inhibiting its ubiquitination. Mechanistically, YTHDF2 stabilized minichromosome maintenance protein 2 (*MCM2*) and *MCM5* transcripts in an m^6^A-dependent manner, thus promoting cell cycle progression and HBV-related HCC tumorigenesis. Moreover, targeting YTHDF2 O-GlcNAcylation by the OGT inhibitor OSMI-1 significantly suppressed HCC progression. Taken together, our findings reveal a new regulatory mechanism for YTHDF2 and highlight an essential role of YTHDF2 O-GlcNAcylation in RNA m^6^A methylation and HCC progression. Further description of the molecular pathway has the potential to yield therapeutic targets for suppression of HCC progression due to HBV infection.

## Introduction

Chronic infection with hepatitis B virus (HBV) has long been recognized as one of the major pathogenic factors in the initiation and development of hepatocellular carcinoma (HCC), contributing to more than half of the HCC incidents worldwide.^1,2^ Recent findings indicate that metabolic reprogramming plays an important role in viral replication and carcinogenesis.^3–5^ Oncogenic viruses promote cancer progression not only by directly integrating viral genes into the host genome, but also by hijacking cellular physiology and metabolism to promote metabolic alterations associated with rapid cell proliferation and tumorigenesis. As a “metabolic virus”, HBV affects numerous hepatic metabolic pathways, including dysregulation of lipid metabolism and enhanced glycolysis. The latter phenomenon is associated with the Warburg phenotype of hepatic cells, which is a collection of cellular features characteristic of tumor cells.^6,7^ A recent study has showed that the hexosamine biosynthesis pathway (HBP), phosphatidylcholine biosynthesis, and nucleotide synthesis are all upregulated during HBV infection.^8^ The HBP converts glucose to UDP-*N*-acetylglucosamine (UDP-GlcNAc), a substrate for O-linked β-N-acetylglucosamine (O-GlcNAc) modification (also known as O-GlcNAcylation), which is catalyzed by O-GlcNAc transferase (OGT) and O-GlcNAcase (OGA).^9,10^ This dynamic O-GlcNAcylation modifies multitude of intracellular proteins, including transcription factors, signaling molecules, and metabolic enzymes. This process also plays an essential role in sustaining important cellular processes such as cellular proliferation, survival, and invasion.

O-GlcNAcylation is a post-translational modification (PTM) that links nutrient flux to gene transcription in virus replication and tumorigenesis.^11,12^ It affects intracellular protein–protein interactions, stability, localization, and activity.^13^ Human T-cell lymphotropic virus type 1 oncoprotein Tax inhibits OGA activity and enhances O-GlcNAcylation of CREB, thus promoting its binding to the viral promoter.^14^ The high-risk human papillomavirus 16 oncogene E6/E7 promotes cervical neoplasm progression through upregulation of OGT and O-GlcNAcylation, which further stabilizes c-MYC via O-GlcNAc modification on Thr58.^15^ Similarly, we found that HBP flux and O-GlcNAcylation levels were markedly elevated upon HBV infection.^16^ Among the host proteins modified by O-GlcNAc, the N^6^-Methyladenosine (m^6^A) reader YTH (YT521-B homology) domain 2 (YTHDF2) attracted our attention. As one of the first identified functional m^6^A readers, YTHDF2 has been widely reported to mediate the stability or translation of m^6^A transcripts.^17–20^ However, the function of O-GlcNAcylation on YTHDF2 has not yet been explored, and its role in HBV pathogenesis remains largely unknown.

m^6^A RNA modification is a dynamic and reversible epigenetic event that prevalent in eukaryotic mRNAs and lncRNAs.^21^ N^6^-methylation is mainly enriched in adenosine, with typical conserved sequence.^22^ It is deposited by the m^6^A methyltransferase complex (“writers”) containing methyltransferase (MTase) complex methyltransferase-like 3 (METTL3), METTL14 and Wilms’ tumor-1-associating protein (WTAP), and removed by demethylases (“erasers”) fat mass and obesity-associated gene (FTO) or AlkB homolog 5 (ALKBH5).^23^ m^6^A can influence the entire RNA life cycle, including RNA splicing, RNA export, RNA translation, and RNA decay, by binding with different “readers”, such as YTHDF2.^18,24,25^ Recent studies have demonstrated that YTHDF2 may play a contradictory role in tumorigenesis and malignant progression of HCC. YTHDF2 promotes the liver cancer stem cell phenotype and metastasis through stabilizing m^6^A modification of *OCT4* transcript.^20^ On the contrary, YTHDF2 also performs as a tumor suppressor and inhibits HCC growth by accelerating the degradation of *EGFR* transcript.^26^ Thus, the specific functions and potential mechanism of the m^6^A reader YTHDF2 in HCC require further exploration.

In this study, we investigated the role of O-GlcNAcylation of the m^6^A reader YTHDF2 in HBV-related hepatocarcinogenesis and progression. We found that YTHDF2 O-GlcNAcylation was elevated upon HBV infection through upregulation of HBP. OGT-mediated YTHDF2 O-GlcNAcylation at Ser263 promoted the mRNA stabilization of *MCM2* and *MCM5* in an m^6^A-dependent manner in HCC. Importantly, our study proposes that targeting OGT-mediated YTHDF2 O-GlcNAcylation may be a novel strategy for the potential treatment of HBV-associated liver cancer.

## Results

### HBV infection enhances YTHDF2 O-GlcNAcylation

In our previous study, we screened putative O-GlcNAcylated proteins in the stable HBV-expressing hepatoma cell line HepAD38 cells (Tet-off) using liquid chromatography-tandem mass spectrometry (LC-MS/MS). In total, 1034 candidates were identified as potential O-GlcNAc-modified proteins. Gene ontology analysis suggested that m^6^A readers, such as YTHDF2/3, hnRNPA2B1, and others, are almost possible to be potentially O-GlcNAcylated (Supplementary Fig. 1a). We focused on the most concerned m^6^A reader YTHDF2 in our subsequent research.

**Fig. 1:**
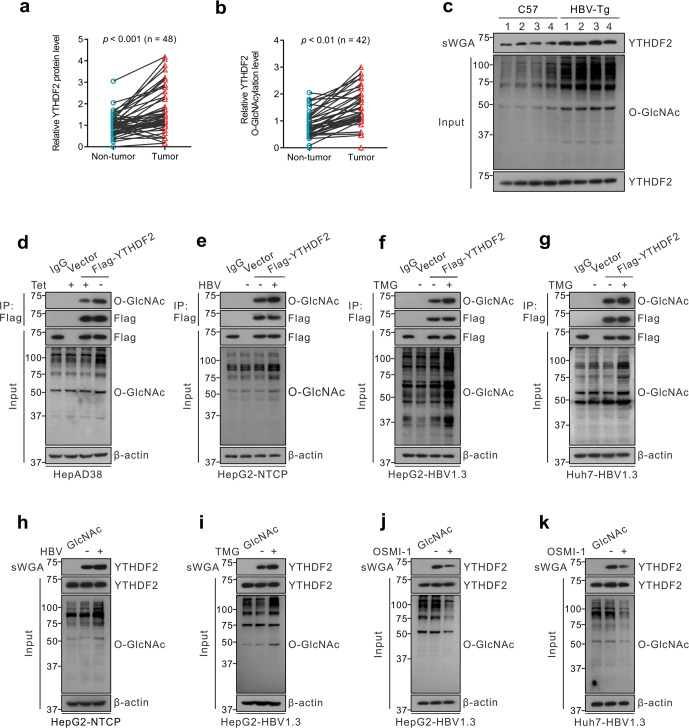
YTHDF2 O-GlcNAcylation is enhanced upon HBV infection. **a** Immunoblots quantitative analysis of YTHDF2 expression in HBV-associated HCC tumors (*n* = 48), *P* < 0.001. **b** Analysis of YTHDF2 O-GlcNAcylation in HBV-associated HCC tumors (*n* = 42) by succinylated wheat germ agglutinin (sWGA) pull-down assays. YTHDF2 O-GlcNAcylation levels were quantified, *P* < 0.01. **c** Analysis of YTHDF2 O-GlcNAcylation in HBV-transgenic mice (*n* = 4), normal C57 mice were used as control. **d**–**g** YTHDF2 Immunoprecipitation (IP) with anti-Flag M2 agarose beads in hepatoma cells transfected with Flag-YTHDF2 or a vector control. Specifically, HepAD38 cells were cultured without tetracycline (tet) (**d**), HepG2-NTCP cells were infected with HBV viruses (**e**), HepG2 cells (**f**) and Huh7 cells (**g**) were infected with AdHBV1.3 (named as HepG2-HBV1.3 and Huh7-HBV1.3) and treated with 25 μΜ Thiamet G (TMG) for 12 h. **h**–**k** sWGA pull-down assays were performed in HepG2-NTCP cells infected with HBV viruses (**h**), HepG2-HBV1.3 cells (**i**, **j**) and Huh7-HBV1.3 cells (**k**) treated with 25 μΜ TMG or 20 μΜ OSMI-1 for 12 h. Western blotting was determined by anti-YTHDF2. All the presented input was adjusted to a similar level for the following IP or sWGA-binding assay

To identify YTHDF2 O-GlcNAcylation in HBV-associated HCC, we first examined YTHDF2 protein expression in 48 paired clinical HBV-associated HCC and normal tissue samples. Immunoblotting showed that YTHDF2 protein levels were markedly higher in HCC tissues than in the corresponding non-tumor tissues (Fig. 1a, *P* < 0.001). Furthermore, we analyzed O-GlcNAcylation of YTHDF2 in paired liver tissues, using succinylated wheat germ agglutinin (sWGA) pull-down assay, a modified lectin that specifically binds to O-GlcNAc on proteins. YTHDF2 O-GlcNAcylation was found to be significantly upregulated in HBV-associated HCC tissues (Fig. 1b, *P* < 0.01). In addition, we extracted four paired liver tissues from HBV-transgenic (Tg) mice and normal C57BL/6 mice and found that both total O-GlcNAcylation and YTHDF2 O-GlcNAcylation levels were markedly increased in HBV-Tg tissues (Fig. 1c, *n* = 4).

To further verify YTHDF2 O-GlcNAcylation, we performed immunoprecipitation (IP) in HBV-replicated hepatoma cell HepAD38 (Tet-off), HBV-infected HepG2-NTCP, and OGA inhibitor Thiamet G (TMG)-treated HepG2-HBV1.3 (AdHBV1.3 infected) and Huh7-HBV1.3 cells (Fig. 1d–g). Flag-tagged YTHDF2 exhibited a distinct O-GlcNAcylation signal in those cells. Moreover, sWGA pull-down assays in hepatoma cells and HEK293 cells indicated that HBV infection or TMG treatment significantly enhanced total O-GlcNAcylation and YTHDF2 O-GlcNAcylation (Supplementary Fig. 1b–g and Fig. 1h, i), whereas the OGT inhibitor OSMI-1 downregulated both processes (Supplementary Fig. 1h, i and Fig. 1j, k). These findings suggest that YTHDF2 O-GlcNAcylation is enhanced after HBV infection or in HBV-associated HCC, and modulated by intracellular O-GlcNAcylation levels.

### Ser263 is the major O-GlcNAcylation site of YTHDF2

**Fig. 2:**
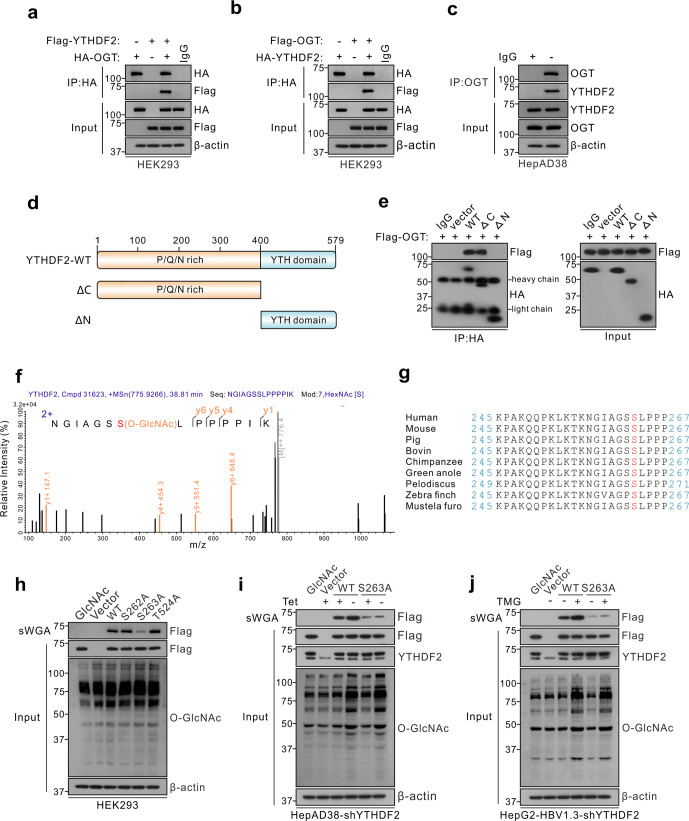
OGT mediates O-GlcNAcylation of YTHDF2 on Ser263. **a**, **b** Co-IP of OGT and YTHDF2 with anti-HA in HEK293 cells co-transfected with Flag (or HA)-YTHDF2 and HA (or Flag)-OGT expression constructs. **c** Co-IP of endogenous OGT and YTHDF2 in HepAD38 cells. **d** Schematic representation of the YTHDF2 constructs. YTHDF2-WT contains two domains, amino-terminal region (1–400aa, ΔC) and YTH domain (401–579aa, ΔN). **e** The interactions between OGT and the full-length or the truncated YTHDF2 (ΔC or ΔN) were determined in HEK293 cells by Co-IP. **f** LC-MS analysis of Flag-YTHDF2 identified residue Ser263 as the YTHDF2 O-GlcNAcylation site. **g** Cross-species sequence alignment of YTHDF2. **h** sWGA pull-down assay with anti-Flag in HEK293 cells. Cells were transfected with vectors encoding Flag-tagged YTHDF2 (WT, S262A, S263A or T524A). **i**, **j** YTHDF2 knockdown was performed in HepAD38 cells (**i**, with or without tet) or HepG2-HBV1.3 cells (**j**) by using lentiviral shRNA. Then cells were transfected with Flag-tagged YTHDF2 (WT or S263A), and subjected to sWGA pull-down assays. All the presented input was adjusted to a similar level for the following IP or sWGA-binding assay

OGT is the sole enzyme responsible for the O-GlcNAcylation of thousands of proteins. To confirm that O-GlcNAcylation of YTHDF2 is mediated by OGT, co-immunoprecipitation (Co-IP) and confocal assays were performed to demonstrate the interaction between OGT and YTHDF2 in HEK293 and HBV-infected hepatoma cells (Fig. 2a–c and Supplementary Fig. 2a). We constructed two YTHDF2 deletion mutants (Fig. 2d) and found that the N-terminal region of YTHDF2 (amino acids 1-400) is required for its interaction with OGT (Fig. 2e). To identify the O-GlcNAcylation site(s) on YTHDF2, Flag-tagged YTHDF2 was immunoprecipitated from HBV-infected HepG2 cells and analyzed by MS. The results showed that Ser263 (S263) was the major O-GlcNAcylation site on YTHDF2 (Fig. 2f). Interestingly, YTHDF2 S263 is well conserved among vertebrates (Fig. 2g). Mutation of Ser263 to Ala (S263A) largely reduced the O-GlcNAc signal compared to the YTHDF2-WT, S262A or T524A mutants in HEK293 cells (Fig. 2h). Furthermore, we established cell lines that stably expressed a short hairpin RNA (shRNA) targeting 3’-untranslated region (UTR) of endogenous *YTHDF2*. Flag-tagged YTHDF2-WT or -S263A were overexpressed in HepAD38-shYTHDF2 cells or HepG2-HBV1.3-shYTHDF2 cells, and the S263A mutant markedly decreased YTHDF2 O-GlcNAcylation, which could not be increased after HBV infection or TMG treatment (Fig. 2i, j). The results of the experiments described above indicate that S263 is the major O-GlcNAcylation site in YTHDF2.

### OGT-mediated O-GlcNAcylation on Ser263 enhances YTHDF2 protein stability by counteracting its ubiquitination

O-GlcNAcylation is involved in many cellular processes and signaling pathways by regulating the localization, stability, and protein–protein interactions of targeted proteins. To explore the effect of O-GlcNAcylation on YTHDF2, endogenous YTHDF2 was examined firstly in HBV-infected HepAD38 and HepG2 cells after treatment with an OGA inhibitor (Supplementary Fig. 2b, c). Immunoblotting showed that YTHDF2 protein expression was greatly increased upon HBV infection and TMG treatment; however, no significant change was observed in *YTHDF2* mRNA levels (Supplementary Fig. 2d). Furthermore, YTHDF2 protein level was significantly downregulated by OGT knockdown, and this inhibition cannot be entirely rescued by HBV infection (Supplementary Fig. 2e), indicating that HBV-induced upregulation of YTHDF2 was mainly dependent on OGT-mediated O-GlcNAcylation. The protein degradation pathways in eukaryotic cells mainly include the ubiquitin-proteasome and lysosome pathways. We found that the proteasome inhibitor MG132 could alone significantly reverse the downregulation of YTHDF2 induced by OSMI-1 treatment (Supplementary Fig. 2f). Therefore, we consider that O-GlcNAcylation may regulate YTHDF2 expression through the ubiquitin-proteasome pathway.

**Fig. 3:**
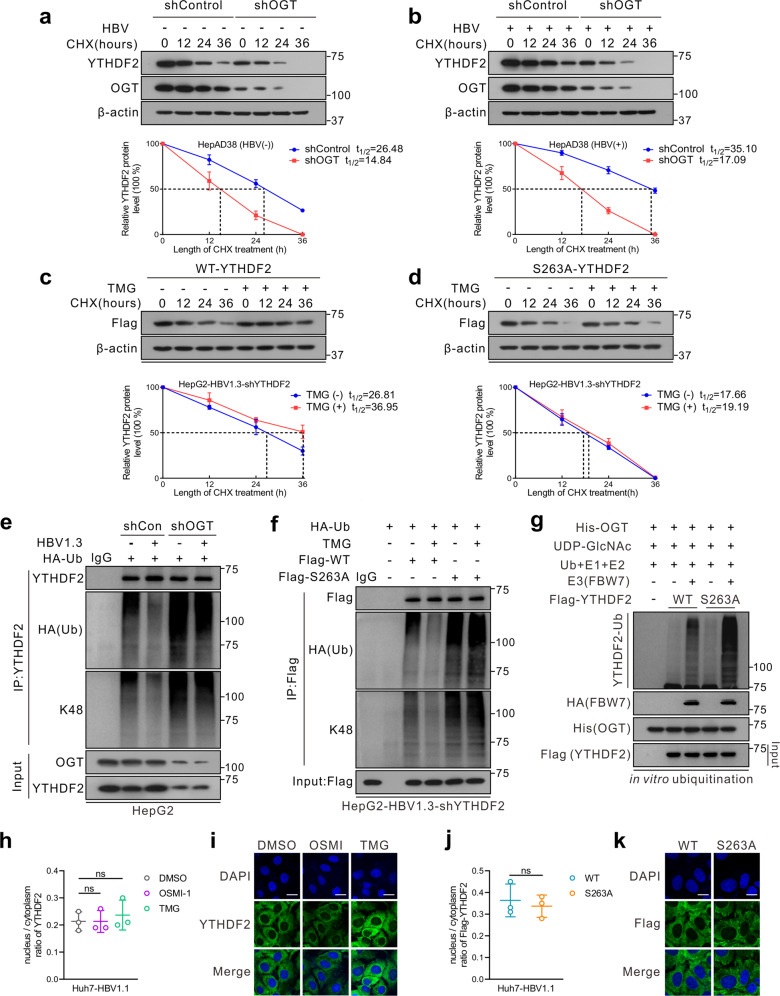
O-GlcNAcylation stabilizes YTHDF2 through suppression of its ubiquitination. **a**, **b** Half-life and quantitative analysis of YTHDF2 in HepAD38 cells treated with (**a**) or without (**b**) tet. Cells were transfected with OGT shRNA lentivirus and treated with 100 μM cycloheximide (CHX) for the indicated time. YTHDF2 levels were analyzed by immunoblotting (*n* = 3, performed in triplicate). **c**, **d** Half-life and quantitative analysis of Flag-YTHDF2 in HepG2-HBV1.3 cells treated with or without 25 μM TMG. HepG2 cells stably with YTHDF2 knockdown (shYTHDF2) were transfected with Flag-tagged YTHDF2-WT (**c**) or YTHDF2-S263A (**d**) and treated with 100 μM CHX (*n* = 3, performed in triplicate). The basal levels of Flag-YTHDF2 expression (WT or S263A) at 0 h were adjusted to a similar level for comparison. Data are expressed as mean ± SD in **a**–**d**. **e**, **f** In vivo YTHDF2 ubiquitination in HepG2 cells in the presence of HA-tagged ubiquitin (HA-Ub). **e** HepG2 cells were transfected with OGT shRNA lentiviral vector, with or without HBV infection; **f** HepG2-HBV1.3-shYTHDF2 cells were transfected with Flag-YTHDF2 (WT or S263A) and treated with 25 μM TMG. Cells were treated with 10 μM MG132 for 4 h to avoid degradation before harvest. After cell lysis, YTHDF2 was immunoprecipitated using anti-YTHDF2 or anti-Flag antibody. **g** In vitro YTHDF2 ubiquitination assay was performed with purified Flag-tagged WT or S263A YTHDF2, His-OGT, SCF^FBW7^ E3 complexes, E1, E2, Ub and UDP-GlcNAc. The reaction products were subjected to immunoblot analysis and YTHDF2 ubiquitination was detected with anti-YTHDF2. **h**–**k** Subcellular localization of YTHDF2 in Huh7 cells transfected with HBV1.1 (pCH9/3091, containing 1.1 copies of the HBV genome) (Huh7-HBV1.1) were determined by immunoblot analysis (**h**, **j**) or immunofluorescence staining (scale bars = 25 μm) (**i**, **k**). Nuclear and cytosolic fractions were immunoblotted with anti-YTHDF2 or anti-Flag, and the nucleus/cytoplasm ratio of YTHDF2 was quantified (*n* = 3, performed in triplicate) (**h**, **j**). Representative images for **h** and **j** were in Supplementary Fig. 3a, b. For **h** and **i**, Huh7-HBV1.1 cells were treated with 25 μM TMG or 20 μM OSMI-1 for 24 h; for **j** and **k**, Huh7-HBV1.1 cells with YTHDF2 knockdown (shYTHDF2) were transfected with Flag-YTHDF2 (WT or S263A)

Subsequently, Flag-tagged YTHDF2 was transfected into HepG2-HBV1.3 cells after treatment with shOGT or shOGA. Cycloheximide (CHX) chase analysis showed that OGT knockdown accelerated the degradation of the YTHDF2 protein, while silencing of OGA decelerated its rate of decay (Supplementary Fig. 2g). In addition, endogenous YTHDF2 protein was more stable after HBV infection in HepAD38 cells, exhibiting a half-life of more than 24 h (Fig. 3a, b). However, knockdown of OGT greatly shortened YTHDF2 lifespan, regardless of HBV infection status. Furthermore, in comparison with YTHDF2-WT, S263A also significantly reduced YTHDF2 half-life; while TMG treatment only extended YTHDF2-WT lifespan but had minimal effect on S263A (Fig. 3c, d). These findings indicate that HBV infection enhances YTHDF2 protein stability through upregulation of its O-GlcNAcylation.

We further investigated the effect of O-GlcNAcylation on YTHDF2 ubiquitination. Treatment with the OGT inhibitor OSMI-1 or shOGT significantly increased total and K48-linked ubiquitination of YTHDF2, while HBV infection had the opposite effect (Supplementary Fig. 2h, i and Fig. 3e). Additionally, compared to YTHDF2-WT, the S263A mutation enhanced YTHDF2 ubiquitination, whereas TMG or OSMI-1 only influenced WT ubiquitination, but had minimal effects on S263A (Fig. 3f and Supplementary Fig. 2j). To further verify the regulation of O-GlcNAcylation on YTHDF2 ubiquitination, an in vitro ubiquitination assay (Fig. 3g) was performed with E3-ubiquitin ligase F-box and WD repeat domain-containing 7 (FBW7), which has been reported to mediate proteolytic degradation of YTHDF2.^27^ When compared to YTHDF2-WT, the S263A mutant significantly increased its ubiquitination level mediated by FBW7. These findings suggest that O-GlcNAcylation of YTHDF2 at Ser263 stabilizes YTHDF2 by counteracting its ubiquitination.

In addition, we assessed the potential effect of YTHDF2 O-GlcNAcylation on its subcellular localization. The nuclear/cytosolic fractionation assay and immunofluorescence assay demonstrated that TMG or OSMI-1 mainly changed YTHDF2 expression, but had minimal effects on its localization (Fig. 3h, i and Supplementary Fig. 3a–f). Moreover, Flag-tagged YTHDF2-WT or S263A was re-expressed in Huh7-shYTHDF2 cells or HepG2-shYTHDF2 cells (Fig. 3j, k and Supplementary Fig. 3a–f). Similarly, no differences were observed in subcellular location between WT and S263A. Considering that YTHDF2 is an important m^6^A reader, we further performed m^6^A Co-IP to explore whether O-GlcNAcylation influences its binding affinity to m^6^A-modified RNA. YTHDF2 consists of a YTH domain that specifically recognizes the m^6^A motif, and we generated a W432A mutant (a known mutation with diminished affinity for m^6^A RNA^17^) as a positive control for the following study. The results showed that treatment of TMG or OSMI-1 had minimal effects on YTHDF2-binding affinity, and there was not much difference between WT and S263A (Supplementary Fig. 3g). These results indicate that O-GlcNAcylation of YTHDF2 enhances YTHDF2 protein stability, but has a minimal effect on its subcellular localization and binding affinity.

### O-GlcNAcylation of YTHDF2 promotes HCC progression

**Fig. 4:**
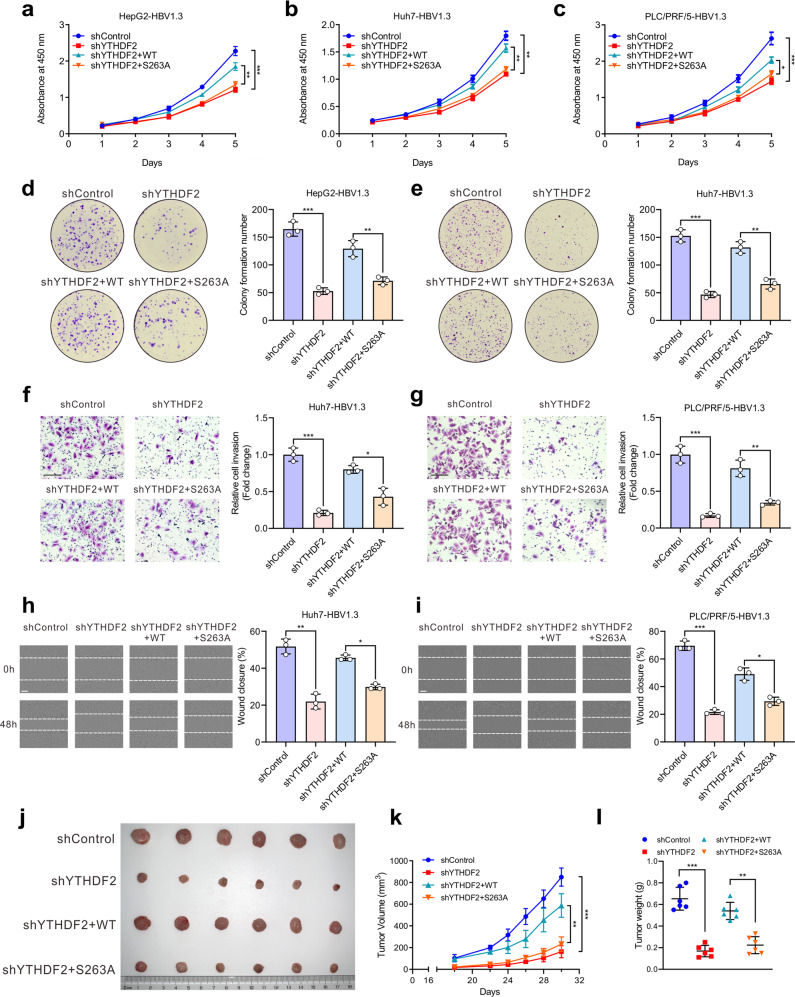
O-GlcNAcylation of YTHDF2 promotes hepatoma cell proliferation, invasion and migration in vitro and in vivo. Cells were transfected with YTHDF2 shRNA lentiviral vector to induce endogenous YTHDF2 knockdown, and subsequently infected with adenoviruses expressing Flag-YTHDF2 (WT or S263A). All hepatoma cells were infected with AdHBV1.3. **a**–**c** Proliferation ability of HepG2-HBV1.3 cells (**a**), Huh7-HBV1.3 cells (**b**) and PLC/PRF/5-HBV1.3 cells (**c**) was detected by CCK-8 assay as indicated (*n* = 3, performed in triplicate). **d**, **e** Colony formation capacity of HepG2-HBV1.3 cells (**d**) and Huh7-HBV1.3 cells (**e**) treated as indicated (*n* = 3, performed in triplicate). **f**, **g** Cell invasion capacity of Huh7-HBV1.3 cells (**f**) and PLC/PRF/5-HBV1.3 cells (**g**) was measured by transwell assay as indicated (*n* = 3, performed in triplicate, bar = 100 μm). **h**, **i** Cell migration capacity of Huh7-HBV1.3 cells (**h**) and PLC/PRF/5-HBV1.3 cells (**i**) was measured by wound-healing assay as indicated (*n* = 3, performed in triplicate, bar = 200 μm). **j**–**l** MHCC-97H cells were treated as indicated and subcutaneously injected into nude mice (*n* = 6 per group). **j** Representative appearance of subcutaneous implantation tumors. **k** and **l** Tumor volume (**k**) and tumor weight (**l**) of implantation tumors. Data are represented as mean ± SD. One-way ANOVA followed by Tukey’s test, *P < 0.05, **P < 0.01, ***P < 0.001

Since YTHDF2 plays an important role in cancer progression and O-GlcNAcylation could enhance its protein stability, we wondered whether O-GlcNAcylation of YTHDF2 is involved in HCC progression. CCK-8 and colony formation assays indicated that depletion of YTHDF2 markedly inhibited cell proliferation in HBV-infected hepatoma cells (Fig. 4a–e and Supplementary Fig. 4a–d), which was rescued by re-expression of YTHDF2-WT but not YTHDF2-S263A. Furthermore, transwell and wound-healing assays also showed that YTHDF2 knockdown decreased the invasion and migration abilities of hepatoma cells. Re-expression of YTHDF2-WT, but not of S263A, obviously increased cell invasion and migration (Fig. 4f–i and Supplementary Fig. 4e). To further explore the role of YTHDF2 in HCC progression in vivo, xenograft tumor experiments were performed by subcutaneously injecting MHCC-97H cells into nude mice. We found that depletion of YTHDF2 greatly inhibited HCC growth, as reflected by the decrease in tumor volume, tumor weight, and immunohistochemical (IHC) staining of Ki67, which was recovered by re-expressing YTHDF2-WT but not YTHDF2-S263A (Fig. 4j–l and Supplementary Fig. 4f). Together, these data show that O-GlcNAcylation of YTHDF2 promotes HBV-related HCC progression.

### Identification of YTHDF2 targets by high-throughput m^6^A-seq and RIP-seq

**Fig. 5:**
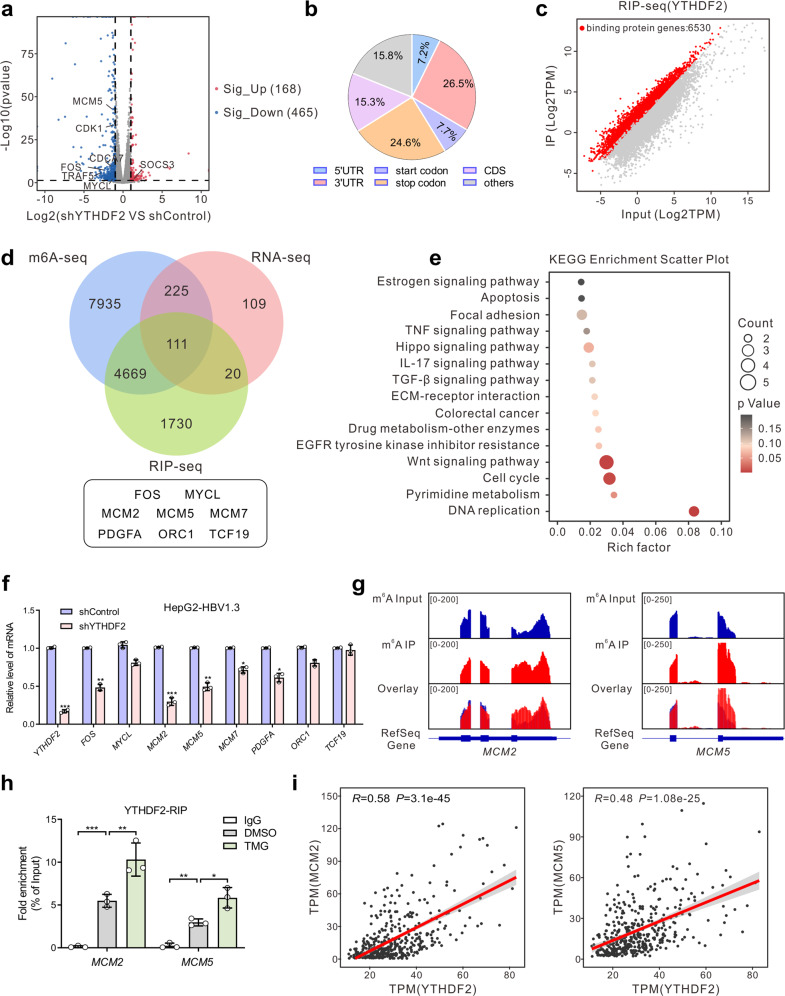
Identification of YTHDF2 targets by high-throughput RNA-seq, m^6^A-seq and RIP-seq. **a** Volcano plot of RNA-seq results in shControl and shYTHDF2 HBV-infected HepG2 cells. Blue and red dots indicate |log_2_FC | ≥ 1 and *P*-value ≤ 0.05. **b** Graphs of m^6^A peak distribution showing the proportion of total m^6^A peaks in the indicated regions in HBV-infected HepG2 cells. **c** Scatter plot of YTHDF2 RIP-seq (IP versus Input) in HBV-infected HepG2 cells. Red dots represent genes that are differentially upregulated or YTHDF2-binding genes, and gray dots represent genes that are not differentially expressed or differentially downregulated. **d** Venn diagram illustrating the overlapped targets of RNA-seq (downregulated upon YTHDF2 knockdown), m^6^A-seq and RIP-seq. **e** KEGG enrichment analysis of overlapped differentially expressed genes (DEGs) identified by RNA-seq, m^6^A-seq and RIP-seq. **f** Relative mRNA levels of initial screening genes in HepG2-HBV1.3 cells identified by RT-qPCR (*n* = 3, performed in triplicate). **g** Distribution of m^6^A peaks across *MCM2* (left) and *MCM5* (right) transcripts. **h** YTHDF2-RIP-qPCR showing the association of *MCM2* or *MCM5* transcripts with YTHDF2 in HepG2-HBV1.3 cells (*n* = 3, performed in triplicate). **i** Correlation analysis between *YTHDF2* and *MCM2* (left), or *YTHDF2* and *MCM5* (right) in TCGA-LIHC cohort (Pearson correlation, *P* < 0.001). Data are represented as mean ± SD. For **f** and **h**, data were analyzed by one-way ANOVA followed by Tukey’s test, *P < 0.05, **P < 0.01, ***P < 0.001

YTHDF2 is one of the major m^6^A readers, that can recognize dynamic m^6^A modifications to regulate the stability and translation status of methylated mRNA.^23^ To identify the potential targets regulated by YTHDF2, RNA sequencing (RNA-seq) was performed in HBV-infected HepG2 cells (Supplementary Fig. 5a). Dysregulated gene expression was caused by YTHDF2 depletion, with 168 elevated and 465 downregulated genes (Fig. 5a), including many oncogenes such as *TRAF5, FOS, MYCL, CDK1*, etc. Subsequently, the m^6^A methylome of HBV-infected HepG2 cells was mapped using m^6^A-seq, and 27,346 m^6^A peaks in 12940 genes were identified. These m^6^A modifications were preferentially enriched in 3’-UTRs (26.5%), stop codons (24.6%) and protein-coding transcripts (15.3%) (Fig. 5b and Supplementary Fig. 5b). Consistent with previous studies,^18^ GGAC [U/A] was identified as the consensus sequence of m^6^A in HepG2 cells (Supplementary Fig. 5c). By combining m^6^A-seq and RNA-seq data, we observed that 336 m^6^A-modified genes were consistently downregulated upon YTHDF2 knockdown (Supplementary Fig. 5d). Gene ontology and KEGG analyses revealed that differentially expressed genes (DEGs) were mainly enriched in cancer-related programs or pathways, such as processes of DNA replication, apoptosis, cell cycle, and the NF-κB signaling pathway (Supplementary Fig. 5e, f). These results indicate that YTHDF2 is a potent regulator of HBV-related HCC in an m^6^A-dependent manner.

To explore YTHDF2 downstream targets, we further conducted RNA immunoprecipitation sequencing (RIP-seq) in HBV-infected HepG2 cells, and found 6530 genes that were directly bound by YTHDF2 (Fig. 5c). After looking for overlaps between YTHDF2 RIP-seq, m^6^A-seq, and RNA-seq data from HBV-infected HepG2 cells, we identified 111 m^6^A-modified genes directly bound by YTHDF2 that were downregulated upon depletion of YTHDF2 (Fig. 5d). In addition, KEGG analysis suggested that DEGs were generally involved in DNA replication and the cell cycle (Fig. 5e). Subsequently, we screened the mRNA of DNA replication-related genes *MCM2* and *MCM5* (*MCM2*/*5*) and found that they were significantly downregulated upon YTHDF2 knockdown in HepG2-HBV1.3 and Huh7-HBV1.3 cells by RT-qPCR (Fig. 5f and Supplementary Fig. 5g). Moreover, m^6^A peak was significantly enriched in the transcripts of *MCM2* and *MCM5* (Fig. 5g). To further validate that *MCM2*/*5* were direct targets of YTHDF2, RIP-qPCR was performed in HepG2 and Huh7 cells with anti-YTHDF2 (Fig. 5h and Supplementary Fig. 5h). The results confirmed the direct interactions between YTHDF2 protein and *MCM2* or *MCM5* mRNA, which were enhanced upon treatment with TMG. Correlation analysis also suggested that *YTHDF2* was positively correlated with *MCM2*/*5* in the TCGA-LIHC database (Fig. 5i). Therefore, we focused on *MCM2/5* for their potential roles in mediating YTHDF2-regulated HBV-associated HCC.

### YTHDF2 O-GlcNAcylation promotes HCC proliferation by preserving the stability of *MCM2* and *MCM5* transcripts in an m^6^A-dependent manner

**Fig. 6:**
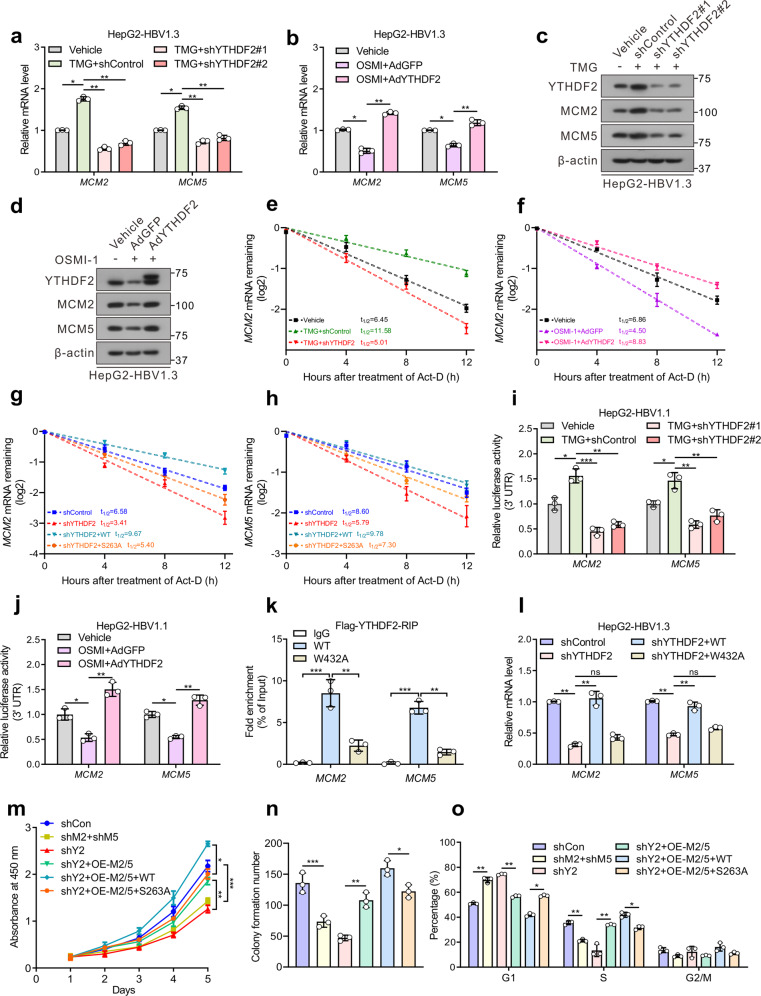
YTHDF2 stabilizes cell cycle-related gene *MCM2* and *MCM5* to promote HCC proliferation. **a**–**d** Relative mRNA levels (**a**, **b**) or immunoblots (**c**, **d**) of *MCM2* and *MCM5* (*MCM2*/*5*). HepG2-HBV1.3 cells (AdHBV1.3 infected) were treated with 25 μM TMG, and followed by transfected with control or two individual YTHDF2 shRNAs (**a**, **c**) or treated with 20 μM OSMI-1, and infected with adenoviruses expressing Flag-YTHDF2 (AdYTHDF2) or control (AdGFP) (**b**, **d**). For **a** and **b**, experiments are performed in triplicate, *n* = 3. **e**, **f** Lifetime of *MCM2* mRNA in HepG2-HBV1.3 cells treated with 25 μM TMG, and followed by transfected with control or YTHDF2 shRNA (**e**) or treated with 20 μM OSMI-1, and infected with AdGFP or AdYTHDF2 (**f**). **g**, **h** Lifetime of *MCM2/5* mRNA in HepG2-HBV1.3-shYTHDF2 cells transfected with Flag-tagged YTHDF2 (WT or S263A). Transcription was inhibited by actinomycin D (5 μg/mL) (*n* = 3, performed in triplicate). **i**, **j** Relative luciferase activity of constructs containing 3’UTR of *MCM2* or *MCM5* in HepG2-HBV1.1 cells (pCH9/3091 transfected) (*n* = 3, performed in triplicate). **k**, **l** HepG2-HBV1.3 cells were transfected with control or YTHDF2 shRNA lentiviral vector, and subsequently transfected with Flag-tagged YTHDF2 (WT or W432A). Flag-RIP-qPCR (**k**) showed the interaction of *MCM2/MCM5* transcripts with YTHDF2. RT-qPCR (**l**) showed the relative *MCM2/5* mRNA levels (*n* = 3, performed in triplicate). **m**–**o** Cell proliferation (**m**), colony formation (**n**) and flow cytometric analysis (**o**) in HepG2-HBV1.3 cells, which were transfected with control (shCon), or YTHDF2 shRNA lentiviral vector (shY2) or MCM2 and MCM5 shRNA lentiviral vectors (shM2 + shM5) to knock down endogenous YTHDF2 or MCM2/MCM5. Then shYTHDF2 groups were subsequently overexpressed with MCM2 and MCM5 (shY2+OE-M2/5), with (or without) overexpression of WT/S263A YTHDF2. All the experiments were performed in HBV-infected cells (*n* = 3, performed in triplicate). Data are represented as mean ± SD. For **a**, **b** and **e**–**o**, data were analyzed by one-way ANOVA followed by Tukey’s test, *P < 0.05, **P < 0.01, ***P < 0.001

To explore the mechanism by which YTHDF2 O-GlcNAcylation regulates *MCM2*/*5* expression, HepG2 and Huh7 cells were treated with the OGA inhibitor TMG, and then by YTHDF2 shRNA. The results showed that TMG treatment increased *MCM2*/*5* mRNA levels, whereas YTHDF2 knockdown reversed the effects of TMG (Fig. 6a and Supplementary Fig. 6a). On the contrary, treatment with the OGT inhibitor OSMI-1 decreased *MCM2*/*5* mRNA levels; this was rescued by the overexpression of YTHDF2 (Fig. 6b and Supplementary Fig. 6b). In addition, the protein levels of MCM2/5 treated as described above showed the similar results (Fig. 6c, d, and Supplementary Fig. 6c, d). In order to investigate whether YTHDF2 regulates the stability of these two transcripts, mRNA stability profiling was performed in HepG2 cells treated with actinomycin D. The median half-lives of *MCM2*/*5* were significantly extended after TMG treatment, but reduced due to shYTHDF2 treatment (Fig. 6e and Supplementary Fig. 6e). In contrast, the decay rates of *MCM2/5* mRNAs were increased upon OSMI-1 treatment, but decreased by overexpression of YTHDF2 (Fig. 6f and Supplementary Fig. 6f). Moreover, the S263A mutant compromised YTHDF2’s ability to stabilize *MCM2/5* mRNAs in HepG2-shYTHDF2 cells (Fig. 6g, h). The luciferase reporter assay further implied that the activities of the 3’ UTRs of *MCM2/5* were increased by YTHDF2 O-GlcNAcylation (Fig. 6i, j). The results collectively suggest that *MCM2*/*5* expressions are regulated by O-GlcNAcylation of YTHDF2.

YTHDF2 consists of a YTH domain that specifically recognizes the m^6^A motif, and the W432A mutation could greatly reduce its affinity for binding methylated RNA.^17^ To further establish the unique role of m^6^A in YTHDF2 function, we performed experiments with the W432A mutant. Flag-RIP-qPCR showed that m^6^A-recognition defective YTHDF2-W432A was unable to enrich *MCM2*/*5* mRNAs, when compared to YTHDF2-WT (Fig. 6k). Furthermore, unlike WT, W432A failed to increase the mRNAs and protein levels of *MCM2*/*5* in HepG2-shYTHDF2 cells (Fig. 6l and Supplementary Fig. 6g, h). Moreover, YTHDF2-W432A had minimal effects on mRNA decay and the 3’ UTR activities of *MCM2/5* (Supplementary Fig. 6i–k). RNA pull-down assay also suggested that the W432A mutation greatly weakened the *MCM2*/*5*-YTHDF2 interaction (Supplementary Fig. 6l). In addition, YTHDF2 failed to upregulate *MCM2*/*5* mRNAs in the main m^6^A writer METTL3-depleted HepG2 cells, while sg*METTL3* reduced the enrichment of *MCM2*/*5* mRNAs by YTHDF2 (Supplementary Fig. 6m–o). These data indicate that YTHDF2 regulates *MCM2/5* mRNAs in an m^6^A-dependent manner.

To determine the role of *MCM2* and *MCM5* in mediating the biological effects of YTHDF2 on HBV-related HCC progression, we generated MCM2/5 overexpressed constructs and shRNA vectors. It has been reported that MCM2/5 are key subunits of the MCM2-7 complex, a replicative helicase important for initiating DNA replication and promoting cell cycle progression, and silencing of MCM2 or MCM5 causes G1/S transition arrest.^28,29^ First, we found that knockdown of MCM2/5 significantly suppressed cellular proliferation, colony formation abilities and caused G1/S arrest, similar to the knockdown of YTHDF2 (Fig. 6m–o and Supplementary Fig. 7a–e). Subsequently, the simultaneous overexpression of MCM2/5-rescued cell proliferation in YTHDF2-depleted HCC cells. Moreover, re-expression of YTHDF2-WT in MCM2/5-rescued cells (YTHDF2-depleted), rather than S263A mutant, further promoted HCC proliferation and cell cycle progression. In addition, invasion and migration abilities were partially recovered by restoration of MCM2/5 and YTHDF2-WT in YTHDF2-depleted Huh7 cells (Supplementary Fig. 7f, g). Collectively, the above data indicate that YTHDF2 O-GlcNAcylation promotes HCC proliferation largely by preserving the stability of *MCM2/5* transcripts in an m^6^A-dependent manner.

### Targeting OGT-mediated YTHDF2 O-GlcNAcylation suppresses HBV-associated hepatocarcinogenesis in vivo

**Fig. 7:**
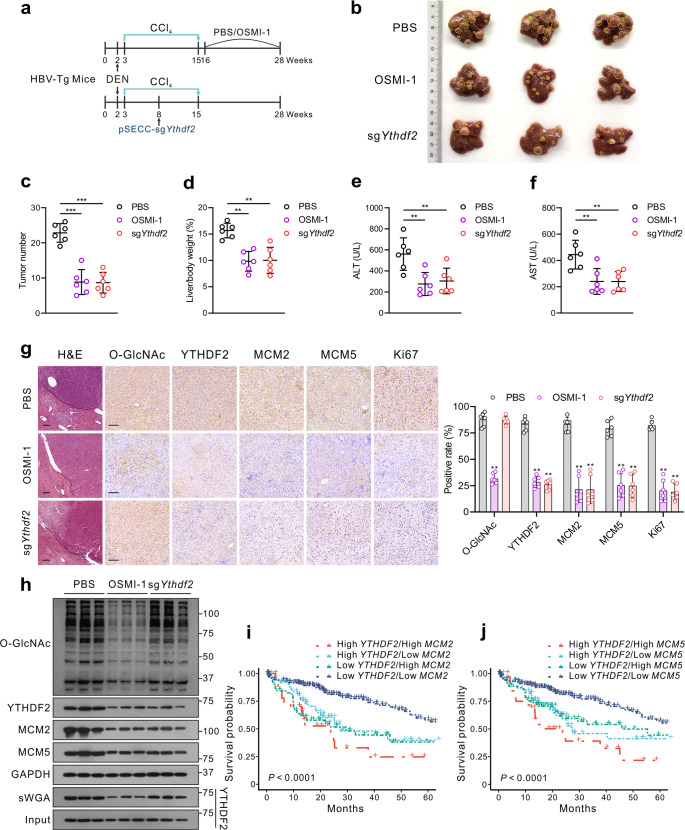
Targeting OGT-meditated YTHDF2 O-GlcNAcylation suppresses HBV-associated hepatocarcinogenesis in vivo. **a** Schematics showing experimental procedures of DEN-induced HBV-transgenic (Tg) mice. **b** Gross appearances of liver samples with tumors. **c**, **d** Tumor nodules numbers (**c**) and liver/body weight (**d**) of HBV-Tg mice, *n* = 6/group. **e**, **f** ALT (**e**) and AST (**f**) levels in mouse serum samples, *n* = 6/group. **g** Representative images of immunohistochemical (IHC) staining of indicated proteins in the liver of HBV-Tg mice and quantification of IHC by using Image J (*n* = 6/group), bar = 100 μm. **h** The indicated proteins expression and YTHDF2 O-GlcNAcylation level in liver tumors of HBV-Tg mice by immunoblots analysis and sWGA pull-down assay. **i**, **j** Kaplan–Meier survival analysis of *YTHDF2* and *MCM2* (**i**) or *YTHDF2* and *MCM5* (**j**) depicting the overall survival (OS) of patients with HCC from the TCGA-LIHC cohort, *P* < 0.0001. Data are represented as mean ± SD. For **c**–**g**, data were analyzed by one-way ANOVA followed by the Tukey’s test, **P < 0.01, ***P < 0.001

Next, we used diethylnitrosamine and carbon tetrachloride (DEN/CCl_4_)-induced HCC models in HBV-transgenic mice and spontaneous HCC mice models (*Alb-Cre Pten*^(flox/flox)^ mice with HBV Ad/rcccDNA transduction) to further validate our results in vivo (Fig. 7a and Supplementary Fig. 8a). HBV infection was identified by serum HBeAg, HBsAg and HBV DNA levels (Supplementary Fig. 8b–e). According to our in vitro results, HBV infection or OGA inhibitor TMG treatment promoted HCC progression by upregulating YTHDF2 O-GlcNAcylation, whereas the OGT inhibitor OSMI-1 had the opposite effect. Therefore, HBV-transgenic mice were treated with DEN and CCl_4_ to induce HBV-associated liver cancer, followed by intraperitoneal injection of OSMI-1 or intravenous injection of pSECC lentiviruses expressing sgRNA targeting *Ythdf2*. Administration of OSMI-1 or pSECC-sg*Ythdf2* resulted in decelerated liver tumorigenesis, as indicated by smaller tumor masses and nodules, as well as lower serum levels of ALT and AST (Fig. 7b-f). Furthermore, IHC staining indicated that YTHDF2, MCM2, MCM5 and Ki67 expressions were greatly decreased upon OSMI-1 treatment or upon injection of pSECC-sg*Ythdf2* (Fig. 7g). Consistent with the in vitro results, total O-GlcNAcylation and YTHDF2 O-GlcNAcylation levels were significantly reduced after OSMI-1 treatment, thereby leading to decreased *MCM2*/*5* mRNA and protein levels (Fig. 7h and Supplementary Fig. 8f, g).

Similar results were observed in HBV-infected spontaneous HCC mice. Specifically, HBV Ad/rcccDNA was intravenously injected to *Alb-Cre Pten*^(flox/flox)^ mice, followed by pSECC-sg*Ythdf2* or OSMI-1 administration as described above. The results indicated that HBV infection upregulated MCM2/5 expression through enhanced YTHDF2 O-GlcNAcylation to accelerate tumor formation, whereas OSMI-1 treatment or pSECC-sg*Ythdf2* exhibited the opposite effect (Supplementary Fig. 8h–o). Moreover, *MCM2*/*5* mRNAs levels, IHC staining and correlation analysis between YTHDF2 O-GlcNAcylation and *MCM2/5* in clinical HBV-associated HCC tissues, proved that *MCM2/5* were significantly upregulated with high expression of YTHDF2 and O-GlcNAcylation (Supplementary Fig. 9a–e). Finally, survival analysis also showed that patients with higher levels of both *YTHDF2* and *MCM2*/*MCM5* had poorer overall survival (Fig. 7i, j). Collectively, these findings suggest that YTHDF2 O-GlcNAcylation increases susceptibility to HBV-related hepatocarcinogenesis via YTHDF2-mediated upregulation of *MCM2/5*. Targeting OGT-mediated YTHDF2 O-GlcNAcylation may be a promising treatment strategy for suppressing HBV-associated HCC.

## Discussion

**Fig. 8:**
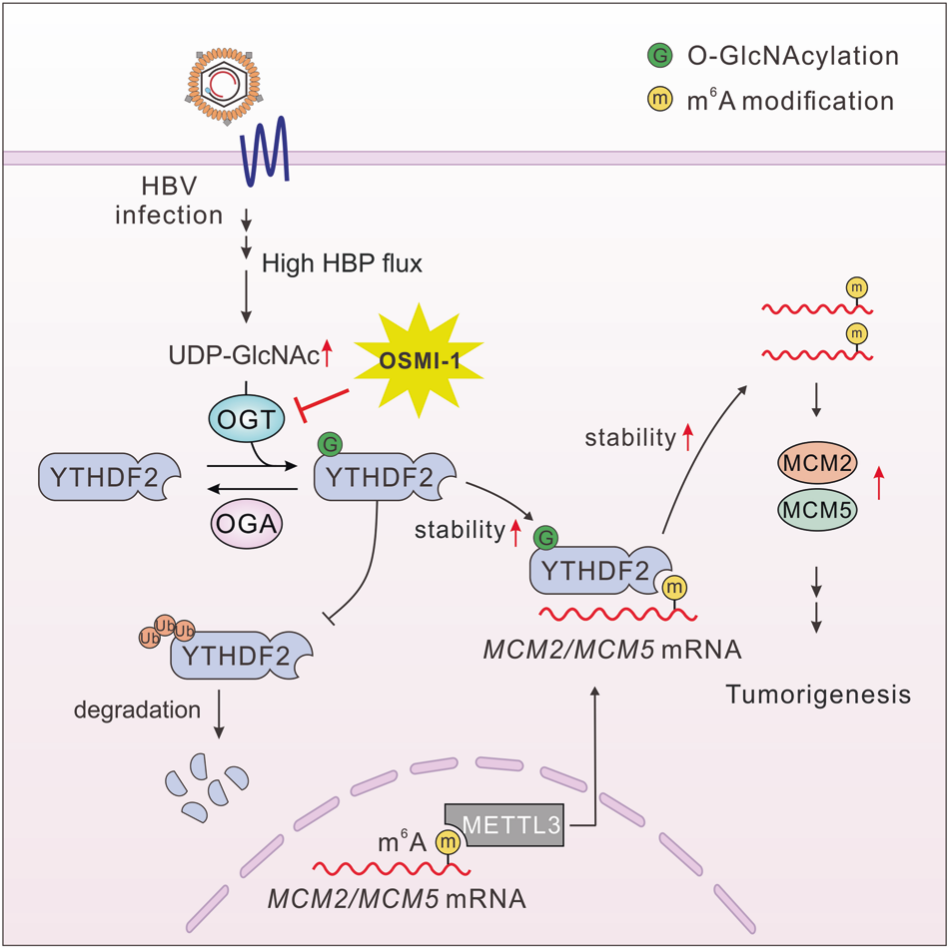
Working model of the study. Schematic depiction of the mechanism underlying O-GlcNAcylation of YTHDF2-mediated HBV-related HCC progression by stabilizing m^6^A-modified *MCM2*/*5*

Accumulating evidence has shown that m^6^A modification widely participates in various biological processes, especially tumorigenesis and cancer progression. As the major reader of m^6^A, YTHDF2 has been reported to promote malignancy of gliomas through facilitating mRNA decay of *UBXN1* and subsequent activation of NF-kB.^30^ In addition, YTHDF2 was found to cooperate with METTL3 and promoted HCC progression by destabilizing mRNA of *SOCS2.*^31^ Previous studies have mostly focused on the role of YTHDF2 in mediating mRNA metabolism and tumor progression through m^6^A methylation; however, the specific functional regulation of YTHDF2, especially post-translational modification, remains largely unexplored. Here, we demonstrate that YTHDF2 O-GlcNAcylation is upregulated during HBV infection and plays a critical role in HCC progression. O-GlcNAcylation at Ser263 maintains its protein stability and oncogenic activity. Targeting YTHDF2 significantly suppresses tumor growth and progression by downregulating *MCM2* and *MCM5*. These findings provide a link between protein O-GlcNAcylation and m^6^A-dependent mRNA regulation in HBV-associated HCC tumorigenesis (Fig. 8).

Recently, post-translational modifications of YTHDF2 have attracted increasing attention. SUMOylation of YTHDF2 at K571 significantly enhanced its direct affinity with m^6^A-modified transcripts, and subsequently led to tumor progression, which resulted from dysregulation of downstream genes.^32^ In addition, phosphorylation of YTHDF2 by EGFR activation at S39/T381, stabilized YTHDF2 protein to accelerate the degradation of *LXRA* and *HIVEP2 mRNA*, which further contributed to glioblastoma tumorigenesis.^33^ Moreover, FBW7 promoted proteolytic degradation of YTHDF2 and consequently stabilized m^6^A-modified pro-apoptotic *BMF* mRNA, thereby suppressing ovarian cancer development.^27^ Our study revealed a previously unrecognized O-GlcNAcylation of YTHDF2 upon HBV infection. Furthermore, we found that YTHDF2 was O-GlcNAcylated at Ser263 and emphasized its oncogenic activity in liver cancer. Our findings highlight the crucial role of O-GlcNAcylation in tumorigenesis, as well as the mechanism responsible for YTHDF2 upregulation in HBV-associated HCC. These findings also further clarify the regulatory network that exists between protein PTM and RNA m^6^A modification in the pathogenesis of HBV-induced HCC.

O-GlcNAcylation has been proved to influence protein’s functional activities, such as stability, transcriptional activity, localization and protein–protein interactions.^34^ O-GlcNAcylation of c-MYC at Thr58 stabilized its protein and inhibited proteasomal degradation by competing with its phosphorylation.^35,36^ Increased YAP O-GlcNAcylation by an OGA inhibitor extended its half-life by inhibiting the SCF^β-TRCP^ E3-ubiquitin ligase.^37^ Here, we found that O-GlcNAcylation of YTHDF2 did not change its intracellular localization or binding affinity to m^6^A-modified RNAs upon HBV infection or treatment with TMG (an OGA inhibitor), but enhanced YTHDF2 protein stability. Loss of O-GlcNAcylation by the S263A mutation shortened YTHDF2 half-life by increasing its K48-linked ubiquitination, thereby promoting its degradation. Furthermore, the S263A mutant largely dampened the carcinogenic role of YTHDF2 in HBV-related HCC. Interestingly, the S263A mutant could not completely abolish O-GlcNAcylation of YTHDF2, implying that other O-GlcNAc sites may exist. Further studies are needed to identify and draw a complete O-GlcNAc modification site map of YTHDF2.

Although RNA and DNA methylation are distinctly regulated, the experience gained from DNA methylation studies cautions against oversimplifying methylation events to univocal carcinogenesis or tumor suppression. As an m^6^A reader, YTHDF2 is reported to play dual roles in HCC,^20,26,31,38,39^ highlighting the complexity of the role of m^6^A modification. Although it is widely known to promote RNA degradation, YTHDF2 also has a positive effect on m^6^A RNA preservation. For example, YTHDF2 was identified as a glioblastoma stem cell-specific m^6^A effector that promotes tumor growth by stabilizing *MYC* transcripts.^40^ In addition, YTHDF2 has been reported to promote *HSP70*, *OCT4* and *6PGD* mRNA translation and participate in cancer progression.^20,41,42^ Interestingly, our results support a similarly non-canonical function of m^6^A methylation. By overlapping data of RNA-seq and m^6^A-seq, we observed that m^6^A modified transcripts tended to be downregulated after silencing of YTHDF2. These results suggest that the exact nature between YTHDF2 and the fate of m^6^A-modified transcripts might be modulated by certain regulators in a cell type-specific manner. More recently, researchers have performed integrative network analysis to identify cell-specific *trans* regulators of m^6^A.^43^ Therefore, the spatial and temporal dynamics regulation of m^6^A methylation may shed new light on the diverse roles of YTHDF2 in different cells or tissues. The underlying mechanisms by which m^6^A selectively functions in specific physiological and pathological processes remain to be elucidated using large-scale m^6^A-seq or single-cell imaging of m^6^A-modified RNA.

In our study, we identified *MCM2* and *MCM5* as YTHDF2 downstream targets using m^6^A-seq and RIP-seq. O-GlcNAcylation of YTHDF2 increased *MCM2* and *MCM5* mRNA stability in an m^6^A-dependent manner, whereas S263A mutation of YTHDF2 failed to extend mRNA lifespan. MCM proteins are indispensable for DNA replication initiation and cell cycle progression, and are thought to be proliferation markers in cancers.^28,44–46^ Our studies showed that MCM2/5 overexpression largely restored YTHDF2 knockdown-induced cell cycle blockage, and upregulated activities of CDKs and cyclins. Moreover, MCM-rescued experiments also showed the potential role of MCM2/5 in YTHDF2-mediated HCC metastasis. These findings suggest that O-GlcNAcylation of YTHDF2 enhances its ability to stabilize *MCM2* and *MCM5* transcripts, thus promoting HBV-related HCC tumorigenesis. However, overexpression of MCM2/5 could not entirely restore tumor growth arrest caused by YTHDF2 depletion, which warrants further studies to identify additional targets of YTHDF2 that participate in HBV-related HCC progression.

In summary, we revealed a link between YTHDF2 O-GlcNAcylation and post-transcriptional regulation of gene expressing upon HBV infection. We demonstrated the regulatory role of HBV-induced O-GlcNAcylation in YTHDF2 overexpression. OGT-mediated YTHDF2 O-GlcNAcylation at Ser263 stabilizes its protein expression and oncogenic activity. Furthermore, we found that YTDHF2 preserved the stability of *MCM2/5* transcripts to promote HBV-related HCC tumorigenesis. HCC progression inhibition by targeting YTHDF2 or YTHDF2 O-GlcNAcylation could be a novel treatment strategy for the intervention of HBV-related HCC.

## Materials and methods

### Ethics statements

The study protocol for clinical patient samples was approved by the Medical Ethics Committee of Chongqing Medical University. Experimental animals were handled according to guidelines approved by the Institutional Animal Care and Use Committee of Chongqing Medical University (license number: 2022050). A pathogen-free environment was provided to keep all mice in the Laboratory Animal Center of the Chongqing Medical University.

### Cell culture

HepAD38, HepG2, and PLC/PRF/5 cells were obtained from the American Type Culture Collection (HB-8065, ATCC, Manassas, VA, USA); MHCC-97H and Huh7 cells were from the Cell Bank of the Chinese Academy of Sciences (Shanghai, China); HEK293 and HEK293T cells were stored in our laboratory. Cells were cultured in Dulbecco’s modified Eagle’s medium (DMEM, SH30243.01, HyClone, Logan, UT, USA) supplemented with 10% fetal bovine serum (FBS, Gibco, Rockville, MD, USA), 100 mg/mL streptomycin (SV30010, HyClone), and 100 units/mL penicillin.

### Animal studies

HBV-transgenic (HBV-Tg) mice (6–8 weeks old, male C57BL/6 mice) were kindly provided by Prof. Ning-shao Xia (Xiamen University, Xiamen, China).^47^ A combination of DEN (50 mg/kg) and CCl_4_ (2 mL/kg, twice per week for 12 weeks) was given to induce HCC in HBV-Tg mice, which were divided into three groups. One group was intravenously injected with pSECC lentiviruses expressing sg*Ythdf2* when mice were aged 6–8 weeks, the other group was intraperitoneally administered with 5 mg/kg OSMI-1 (HY-119738, MedChemExpress (MCE), Monmouth Junction, USA) twice a week for 12 weeks, and the third group was a control group (injection of PBS and pSECC-sgControl lentiviruses). At 28 weeks of age, mice were euthanized for subsequent assessment.

*Alb-Cre Pten*^(flox/flox)^ mice were kindly provided by Prof. Yu-jun Shi (Sichuan University, Sichuan, China). A total of 1.5 × 10^9^ plaque-forming units (PFU) HBV Ad/rcccDNA was intravenously injected to Alb-Cre mice (aged 6–8 weeks, male), followed by administration with pSECC lentiviruses expressing sg*Ythdf2* or OSMI-1 for 18 weeks as described above. At 40 weeks of age, the mice were sacrificed for the relevant experiments.

For the xenograft implantation model, 2 × 10^6^ MHCC-97H cells were subcutaneously injected into the flank of nude mice. Cells were infected with shYTHDF2 lentivirus, followed by AdYTHDF2-WT or AdYTHDF2-S263A. The tumor volume (V) was calculated as follows: *V* [cm^3^] = (length [cm]) × (width [cm]) × (width [cm])/2. Mice were euthanized after implantation for 4–6 weeks, and tumor tissues were excised for analysis.

### Clinical specimens

HBV-associated HCC samples and paired, adjacent normal liver tissues were obtained from the Second Affiliated Hospital of Chongqing Medical University between 2015 and 2020, approved by the Institutional Review Board of Chongqing Medical University.

### Plasmid constructs

The full-length cDNA of Human YTHDF2 (NM_016258.3), OGT (NM_181672.2), FBW7 (NM_033632.3), the C-terminal and N-terminal truncated mutants of YTHDF2 (containing amino-terminal region (1–400aa) and YTH domain (401–579aa), respectively) were amplified by PCR and subcloned into the pBudCE4.1-3HA or pSEB-3Flag. YTHDF2-S262A, YTHDF2-S263A, YTHDF2-T524A, YTHDF2-W432A were constructed by overlapping PCR. Human MCM2 (NM_004526.4) and MCM5 (NM_006739.4) were amplified and subcloned into the pAdTrack-TO4 vector. All the specific primers are provided in Supplementary Table 1.

### Antibodies

Antibodies against YTHDF2 (ab220163), O-GlcNAc (ab2739), OGA (ab124807), OGT (ab96718), K48 (ab271911) and p-Rb (ab173289) were obtained from Abcam (Cambridge, UK). Antibodies against MCM2 (10513-1-AP), MCM5 (11703-1-AP), and β-Tubulin (66240-1-Ig) were obtained from Proteintech (Rosemont, IL, USA). Antibody against HA (26183) was from Invitrogen (Carlsbad, CA, USA). Antibody against FLAG (F3165) was from Sigma-Aldrich (Germany). Antibody against H3 (H0164) was from Millipore (Massachusetts, USA). Antibodies against Rb (A16966), p21 (A21749), cyclinD1 (A2708) and CDK4 (A0366) were from ABclonal Technology (Wuhan, China). Antibodies against β-actin (TA-09), Goat rabbit IgG/TRITC, secondary (ZF-0316), and Goat mouse IgG/FITC, secondary (ZF-0312) were purchased from ZSGB-BIO (Beijing, China). Antibody against OGT (sc-74546, for immunofluorescence) was from Santa Cruz (CA, USA).

### YTHDF2 O-GlcNAcylation site mapping

Liquid chromatography-tandem mass spectrometry (LC-MS/MS) was performed to identify the YTHDF2 O-GlcNAcylation sites, as previously described.^48^ In brief, HepG2-HBV1.3 cells were overexpressed with Flag-YTHDF2, and then YTHDF2 was immunoprecipitated and stained with Coomassie blue. Gel containing YTHDF2 was excised and entrusted to the Shanghai Applied Protein Technology Co., Ltd (Shanghai, China) for analysis. Potential O-GlcNAcylation sites included Ser262, Ser263 and Thr524.

### YTHDF2 protein stability assay

For stability detection of endogenous YTHDF2 protein, HepAD38 cells were treated with 100 μM cycloheximide (CHX, MCE, Monmouth Junction, NJ, USA) and collected at 0, 12, 24 and 36 h. For protein stability detection of exogenous YTHDF2 (WT and S263A mutant), HepG2-shYTHDF2 cells were re-expressed with Flag-WT or Flag-S263A mutant, respectively. Then, cells were collected at 0, 12, 24, and 36 h following 100 μM CHX treatment. The basal levels of Flag-YTHDF2 expression (WT or S263A) at 0 h were adjusted to a similar level for comparison. YTHDF2 protein expression was examined with anti-YTHDF2 or anti-FLAG antibody (Sigma-Aldrich, Germany).

### Immunoprecipitation (IP)

Cells were overexpressed with Flag-YTHDF2, then harvested with lysis buffer (50 mM Tris-HCl, pH 7.4, 1 mM EDTA, 150 mM NaCl, and 1% Triton X-100) containing 1× Protease Inhibitor Cocktail (4693159001, Roche, Switzerland) and 1× Phosphatase Inhibitor (Beyotime Biotechnology). After centrifugation, the supernatants were incubated with anti-FLAG M2 affinity gel (A2220, Sigma-Aldrich, Germany) overnight at 4 °C. For Co-IP, cells were co-transfected with HA-tagged or Flag-tagged OGT and YTHDF2 full-length, or truncated mutants ΔC or ΔN. Pre-cleared cell lysates were incubated with anti-HA antibody overnight at 4 °C, and subsequently incubated with protein A/G agarose beads (Millipore, USA) for 4 h. Immunoprecipitates were washed and immunoblotted with the indicated antibodies, respectively.

### In vivo ubiquitination assay

Cells were transfected with HA-Ubiquitin (HA-Ub) or co-transfected with HA-Ub and Flag-tagged YTHDF2 (WT or S263A). MG132 (10 μM) was applied to prevent degradation before harvesting. At 48 h post-transfection, cells were harvested with lysis buffer containing 1% SDS and boiled for denaturation. After immunoprecipitated with anti-Flag or anti-YTHDF2 antibodies, the supernatants were incubated with protein A/G agarose beads for 4 h. Then beads-captured complexes were washed four times and analyzed by Western blotting. YTHDF2 ubiquitination levels were examined with anti-K48 and anti-HA antibodies.

### In vitro ubiquitination assay

In vitro ubiquitination assay was performed as described previously.^49,50^ Briefly, HEK293 cells were transfected with Flag-tagged YTHDF2 (WT or S263A) to purify various YTHDF2 by anti-FLAG M2 affinity gel. To purify the SCF^FBW7^ E3 ligase complex, HEK293 cells were transfected with plasmids encoding HA-FBW7, Myc-Cullin-1, Myc-SKP1, and Myc-RBX1 (obtained from Tsingke Biotechnology Co., Ltd, Beijing, China). The SCF^FBW7^ E3 complexes were purified from the whole cell lysates by HA affinity precipitation. Afterward, cell-purified Flag-YTHDF2, SCF^FBW7^ complexes and bacterially purified His-OGT were incubated with E1, E2, ubiquitin, ATP and UDP-GlcNAc (HY-112174, MCE, USA) in the reaction buffer (20 mM Tris-HCl pH = 7.4, 5 mM MgCl_2_, 1 mM DTT) according to manufacturer’s instructions (Abcam, ab139469). The reaction was performed at 37 °C for 2 h and stopped by 2×SDS-PAGE buffer. After that, the complexes were immunoblotted with anti-YTHDF2 antibody to detect YTHDF2 ubiquitination.

### sWGA pull-down assay

Human and mouse liver tissues and hepatoma cells were lysed in lysis buffer (125 mM NaCl, 50 mM Tris pH 7.4, 5 mM EDTA, and 0.1% NP-40) containing protease and phosphatase inhibitors. The supernatant was denatured in glycoprotein denaturing buffer and digested with PNGase (P0704S; New England Biolabs, USA) to remove N-linked glycoproteins. After centrifugation, the supernatants were incubated with sWGA-conjugated agarose beads (AL-1023S, Vector Laboratories, Burlingame, USA) overnight at 4 °C. Precipitated complexes were washed and subjected to immunoblotting with anti-YTHDF2 or anti-Flag antibodies. All the presented input was adjusted to a similar level for the following sWGA-binding assay.

### Immunofluorescence (IF)

Cell slides were fixed with 4% paraformaldehyde for 25 min, and then incubated with indicated primary antibody overnight. Subsequently, the slides were incubated with fluorescence-labeled secondary antibody, and nuclear staining was mounted with DAPI (10236276001, Roche Diagnostics GmbH, Mannheim, Germany). Images were taken using a Leica confocal microscope (Leica TCS SP8, Leica Microsystems, Wetzlar, Germany).

### mRNA stability assay

HepG2 cells were treated with 5 μg/mL actinomycin D (S8964, Selleck, TX, USA) for 0, 4, 8, and 12 h, and total RNA was extracted using the TRIzol. Quantitative real-time PCR (RT-qPCR) was performed to analyze mRNA levels. The specific primers are provided in Supplementary Table 1. The mRNA half-life (*t*_1/2_) was calculated using the following equation, as previously described: *t*_1/2_ = In 2/*k*_decay._^33^

### RNA pull-down assay

HepG2-HBV1.3 cells were transfected with Flag-YTHDF2 (WT, S263A and W432A). Pull-down assays were carried out by incubating biotin-*MCM2* or biotin-*MCM5* (synthesized by GeneCreate Biological Engineering Co., Ltd, Wuhan, China) with the HepG2 cell lysates for 0.5 h at 25 °C. The reaction products were precipitated using Pierce™ streptavidin agarose beads, followed by immunoblotting with anti-YTHDF2 antibodies.

### RNA immunoprecipitation and sequencing (RIP and RIP-seq)

RNA immunoprecipitation was performed using the Magna RIP™ RNA-Binding Protein Immunoprecipitation Kit (17-700, Millipore, USA), according to manufacturer’s instructions. Briefly, the cells were lysed with RIP lysis buffer and prepared by freeze-thawing. After centrifugation, the supernatant was incubated with anti-Flag or anti-YTHDF2 antibody conjugated protein A/G magnetic beads for 4–6 h. Next, immunoprecipitates were eluted and subjected to proteinase K treatment, followed by RNA extraction using TRIzol. The relative interaction between YTHDF2 and target RNA was determined by RT-qPCR and normalized to input. For RIP-seq, rRNAs were depleted using the NEBNext rRNA Depletion Kit (New England BioLabs). cDNA libraries were produced by employing NEBNext UltraRNALibrary Prep Kit for Illumina (New England BioLabs) and sequenced on Illumina Novaseq™ 6000 platform at LC-BIO Technology Co., Ltd. (Hangzhou, China) following the vendor’s recommended protocol.

### Methylated RNA immunoprecipitation sequencing (m^6^A-seq)

m^6^A-Seq was performed by LC-Bio Technology Co., Ltd. (Hangzhou, China), according to manufacturer’s instructions. Total RNA from HBV-infected HepG2 cells was isolated, purified and subjected to m^6^A RNA immunoprecipitation. Eluted m^6^A-containing fragments (IP) and untreated input control fragments were converted to the final cDNA library following strand-specific library preparation using the dUTP method. The average insert size for the paired-end libraries was approximately 100 ± 50 bp. Paired-end sequencing (PE150) was performed on an Illumina Novaseq™6000 platform.

### Sequencing data analysis

All sequencing analyses were performed with the help of LC-Bio Technology Co., Ltd and deposited at the Gene Expression Omnibus (GEO) repository under accession number GSE200261. We used hisat2 software (v2.0.4) to map high quality trimmed reads to the genome of Homo sapiens (Version: GRCh38) with default parameters. For m^6^A-seq, peak calling and different peak analyses were performed using the R package exomePeak2, and peaks were annotated by intersection with the gene architecture using the R package ANNOVAR. MEME and HOMER were used for de novo and known motif finding, followed by localization of the motif with respect to the peak summit. StringTie was used to determine expression levels for all transcripts and genes from input libraries by calculating FPKM (total exon fragments /mapped reads (millions) × exon length (kB)). Differentially expressed transcripts and genes were selected with log2 (fold-change) ≥1 or log2 (fold-change) ≤ –1 and *P*-value < 0.05, using the R package edgeR. For RIP-seq, RPKM (reads per kilobase per million reads) was used to measure the expression of all genes. The RIP targets were defined as different genes of IP/Input with log2 (fold-change) ≥1 and *P*-value < 0.05.

### Statistical analysis

Data are shown as mean ± SD. Statistical analyses were performed with GraphPad Prism 7 (GraphPad Software Inc.). Student’s *t*-test or paired *t*-test was used for the comparison of two groups. One-way ANOVA followed by Tukey’s test was used for all other variables. Pearson’s correlation coefficient (R) was used for the comparison of linear correlations. *P*-value < 0.05 was regarded as significant. *P < 0.05, **P < 0.01, ***P < 0.001.

## Supplementary information

Supplementary Information

## Data Availability

Full details are available in the manuscript and the supplementary materials. All the raw data and processed files have been deposited in the Gene Expression Omnibus (http://www.ncbi.nlm.nih.gov/geo) under accession number GSE200261. The Cancer Genome Atlas (TCGA) PanCancer Atlas dataset referenced during the study are available in a public repository from the cBioPortal website (https://www.cbioportal.org/).
