# Supplementary material for: O-GlcNAcylation of YTHDF2 promotes HBV-related hepatocellular carcinoma progression in an N6-methyladenosine-dependent manner
Source: Signal Transduct Target Ther. 2023 Feb 10;8:63. doi: 10.1038/s41392-023-01316-8 (PMC9918532; doi:10.1038/s41392-023-01316-8)
Supplement: Supplementary file 1 — Supplementary Information [file 41392_2023_1316_MOESM1_ESM.docx]

**Supplementary Information**

**O-GlcNAcylation of YTHDF2 promotes HBV-related hepatocellular carcinoma progression in an m^6^A-dependent manner**

Yang Yang^1,#^, Yu Yan^1,#^, Jiaxin Yin^1,#^, Ni Tang^1,#^, Kai Wang^1,#^, Luyi Huang^1^, Jie Hu^1^, Zhongqi Feng^1^, Qingzhu Gao^1^, Ailong Huang*^1^

Correspondence to: Ailong Huang (ahuang@cqmu.edu.cn).

**This PDF file includes:**

Supplementary Materials and Methods

Figures. S1-S9

Table S1

**Supplementary** **Materials and Methods**

**Genomic editing with sgRNA**

Genomic mutations in cells or mice were generated using the CRISPR/Cas9 system as previously described.^1,2^ sgRNA sequences target Human METTL3 or Mouse *Ythdf2* were designed and inserted into the lentiCRISPRv2 (provided by Prof. Ding Xue from the Tsinghua University, Beijing, China) or pSECC (Addgene, catalog 60820) vector using the BsmBI restriction enzyme, respectively. Non-targeting sgRNA sequences that do not recognize any sequence in the human or mouse genome was used as a negative control (sgControl). All the sgRNA target sequences used are listed in Supplementary Table 1.

**Virus production and HBV infection**

For HepAD38 cells, 1 µg/mL tetracycline (A100422, Sangon Biotech, Shanghai, China) was added to suppress HBV transcription. Other hepatoma cells were infected with adenovirus AdHBV1.3, which was provided by Prof. Michael Nassal (University Hospital Freiburg, Freiburg, Germany) or transfected with pCH9/3091 (containing 1.1 copies of the HBV genome) using Lipofectamine 3000 (15338030, Invitrogen, Carlsbad, CA, USA), to sustain all processes of HBV replication. HepG2 cell stably expressing sodium taurocholate co-transporting polypeptide (HepG2-NTCP) was a gift from Prof. Ning-shao Xia (Xiamen University, Xiamen, China) and was infected with HBV virus as previously described.^3^ Briefly, HepG2-NTCP cells were infected with concentrated HBV viral particles derived from supernatants of HepAD38 cells at 1000 genome equivalents (GE) per cell in the Williams’ E media containing 5% PEG 8000 (IP9120, Solarbio) for 24 h. After removing the virus-containing medium, the cells were maintained in DMEM before harvest.

**shRNA constructs**

The small double-strand hairpin RNAs (shRNAs) targeting YTHDF2 were designed and cloned into the Hpa I and Xho I sites of pLL3.7 lentivirus vector (kindly provided by Prof. Bing Sun, Center for Excellence in Molecular Cell Science, Chinese Academy of Sciences, Shanghai, China). Similarly, shRNAs targeting MCM2 or MCM5 were cloned into the pLL3.7 vector. A negative control construct (shControl) was also generated. Oligos are listed in Supplementary Table 1.

**Adenovirus production**

The full-length cDNA fragment of YTHDF2 (NM_016258.3) or S263A (YTHDF2 mutant) was inserted into the pAdTrack-TO4 vector (from Dr. Tong-Chuan He, University of Chicago, USA). The adenoviral recombinant pAdYTHDF2 (AdWT and AdS263A) were generated using the AdEasy system as described previously.^4^ An analogous adenovirus expressing GFP (AdGFP) was used as control.

**Lentivirus production and transduction**

Lentiviral production and transduction were performed as previously described.^5^ Briefly, the lentiviral vectors were co-transfected with packaging vectors psPAX2 and pMD2.G into 293T cells using Lipofectamine 3000. Viruses in the supernatants were collected and filtered at 48 h after transfection. Target cells were infected with lentiviruses supplemented with 5 μg/mL polybrene.

**Western blotting**

Protein lysates of cells or liver samples were extracted with cell lysis buffer (P0013, Beyotime Biotechnology, Shanghai, China) containing 1 mM phenylmethanesulfonyl fluoride (Beyotime Biotechnology). Equal volumes of protein samples were separated by 10% SDS-PAGE and electro-transferred to PVDF membranes (IPVH00010, Merck Millipore, Billerica, MA, USA). The immunoblots were probed with the indicated antibodies. Proteins bands were visualized with Super Signal West Pico Chemiluminescent substrate Kits (Bio-Rad, Hercules, CA, USA). Quantification of bands in Western blotting were performed using Image-Pro Plus software (Media Cybernetics, Rockville, Maryland, USA).

**RNA extraction, reverse transcription PCR, and quantitative real-time PCR**

Total RNA was extracted from clinical specimens and HCC cell lines with TRIzol reagent (Invitrogen, Carlsbad, CA, USA), and reverse transcribed using PrimeScript™ RT Reagent Kit with gDNA Eraser (RR047A, TaKaRa, Tokyo, Japan) following the manufacturer’s instructions. Quantitative real-time PCR (RT-qPCR) was performed to quantify mRNA levels using the SYBR Green qPCR Master Mix (1725121, Bio-Rad, California, USA). The specific primers used are shown in Supplementary Table 1. All the samples were analyzed in triplicate.

**Quantification of HBV DNA by RT-qPCR**

HBV DNA in mice serum was extracted using TIANamp Virus DNA kit according to manufacturer’s instructions. The isolated HBV DNA was subjected to RT-qPCR using SYBR Green qPCR Master Mix. The plasmid pCH9/3091 (containing 1.1 copies of HBV genome) served as a template for the standard curve. The primers are listed in Supplementary Table 1.

**YTHDF2-bound m^6^A RNA detection by Co-IP**

The binding of YTHDF2 with endogenous m^6^A-modified RNA was detected by Co-IP as previously described.^6,7^ Cells were transfected with Flag-tagged YTHDF2 (WT, S263A or W432A). 48 h post-transfection, cells were UV-crosslinked before collection, and then lysed with lysis buffer (50 mM Tris-HCl pH 7.4, 150 mM NaCl, 1% NP-40 and 100 U/mL RNase inhibitor and Protease inhibitor cocktail). YTHDF2 was immunoprecipitated with anti-FLAG M2 affinity gel. Specially, the immunoprecipitation complex was washed twice with high-salt buffer (50 mM Tris–HCl pH 7.4, 300 mM NaCl), followed by two additional washes with low-salt buffer (50 mM Tris–HCl pH 7.4, 150 mM NaCl). The amount of YTHDF2-bound m^6^A RNAs was detected by Western blot with anti-m^6^A antibody.

**Cell proliferation and colony formation assays**

Cells were seeded in 96-well plates at a density of 1 × 10^3^ cells/well and cultured for 5 days. Cell proliferation capability was measured by Cell Counting Kit-8 (CCK-8) assay (TP1197, TargetMol, Wellesley Hills, MA, USA). For colony formation assay, 2 × 10^3^ cells were seeded in 6-well plates and cultured for 2 weeks. Colonies were stained with 0.04% crystal violet, photographed, and counted in triplicate.

**Wound-healing assay**

Cells were cultured in 96-well plates, and wounds were created using WoundMaker™ on the cell surface. After 36 h or 48 h, the wound areas were recorded by the IncuCyte ZOOM Live-Cell Imaging system (Essen BioScience, Ann Arbor, MI, USA).

**Transwell migration assay**

Cell invasion was assessed using Cell Culture Insert (FALCON, USA). Cells were replete onto the upper chamber with serum free medium. The lower chamber was replete with medium containing 10% FBS. The invasive cells were stained with crystal violet and quantified (3 random 200× fields per well) under Axio Imager A2 (ZEISS, Germany).

**Cell cycle analysis**

The cells were fixed with 70% alcohol at 4 °C overnight, and resuspended in PBS with propidium iodide and RNase A for 30 min before application to flow cytometry assay (FACS Calibur; BD Biosciences, Franklin Lakes, NJ, USA), as previously described.^4^

**Luciferase reporter assay**

The 3’ UTR of MCM2 and MCM5 was amplified by PCR according to their m^6^A modification sites, and subcloned into pGL3-Basic vector. Cells were transfected with pGL3-Basic luciferase reporter plasmids containing 3’ UTR of MCM2 or MCM5 and pRL-TK (an internal control) using Lipofectamine 3000. At 48 h post transfection, cells were harvested and measured for luciferase activity using the Dual Luciferase Assay Kit (E1910, Promega, Madison, WI, USA). All experiments were performed at least three times and expressed as mean ± SD.

**Immunohistological (IHC) staining**

Human or mouse liver tissues were fixed in 4% paraformaldehyde (E672002, Sangon Biotech) and embedded in paraffin according to standard procedures. The sections were incubated with the indicated primary antibodies overnight at 4 °C. Subsequently, the slides were incubated with secondary anti-rabbit or anti-mouse IgG (PV-9000, ZSGB-BIO, Beijing, China) and visualized using 3, 3′-diaminobenzidine (ZLI-9019, ZSGB-BIO, Beijing, China). Stained slides were scanned with a Pannoramic Scan 250 Flash or MIDI system, and images obtained by using Pannoramic Viewer 1.15.2 (3DHistech, Budapest, Hungary).

**RNA sequencing**

Total RNA was isolated from YTHDF2 knockdown (shYTHDF2) or control (shControl) HBV-infected HepG2 cells using the TRIzol reagent. RNA-seq and initial analyses were conducted by LC-Bio Technology Co., Ltd. using Illumina 6000 and 2 × 150 bp paired-end sequencing. The sequence results were obtained as FPKM (fragments per kilobase of exon per million reads) for each transcript.

**REFERENCES**

1. Gao, Q. *et al.* SLC27A5 deficiency activates NRF2/TXNRD1 pathway by increased lipid peroxidation in HCC. *Cell Death Differ.* **27**, 1086–1104 (2020).

2. Sánchez-Rivera, F. J. *et al.* Rapid modelling of cooperating genetic events in cancer through somatic genome editing. *Nature* **516**, 428–431 (2014).

3. Chen, Y. *et al.* APOBEC3B edits HBV DNA and inhibits HBV replication during reverse transcription. *Antiviral Res.* **149**, 16–25 (2018).

4. Tuo, L. *et al.* PCK1 negatively regulates cell cycle progression and hepatoma cell proliferation via the AMPK/p27Kip1 axis. *J. Exp. Clin. Cancer Res.* **38**, 50 (2019).

5. Zhao, G. *et al.* Cullin5 deficiency promotes small-cell lung cancer metastasis by stabilizing integrin β1. *J. Clin. Invest.* **129**, 972–987 (2019).

6. Du, Y. *et al.* SUMOylation of the m6A-RNA methyltransferase METTL3 modulates its function. *Nucleic Acids Res.* **46**, 5195–5208 (2018).

7. Alarcón, C. R., Lee, H., Goodarzi, H., Halberg, N. & Tavazoie, S. F. N6-methyladenosine marks primary microRNAs for processing. *Nature* **519**, 482–485 (2015).

**Supplementary Figures and Figure legends**


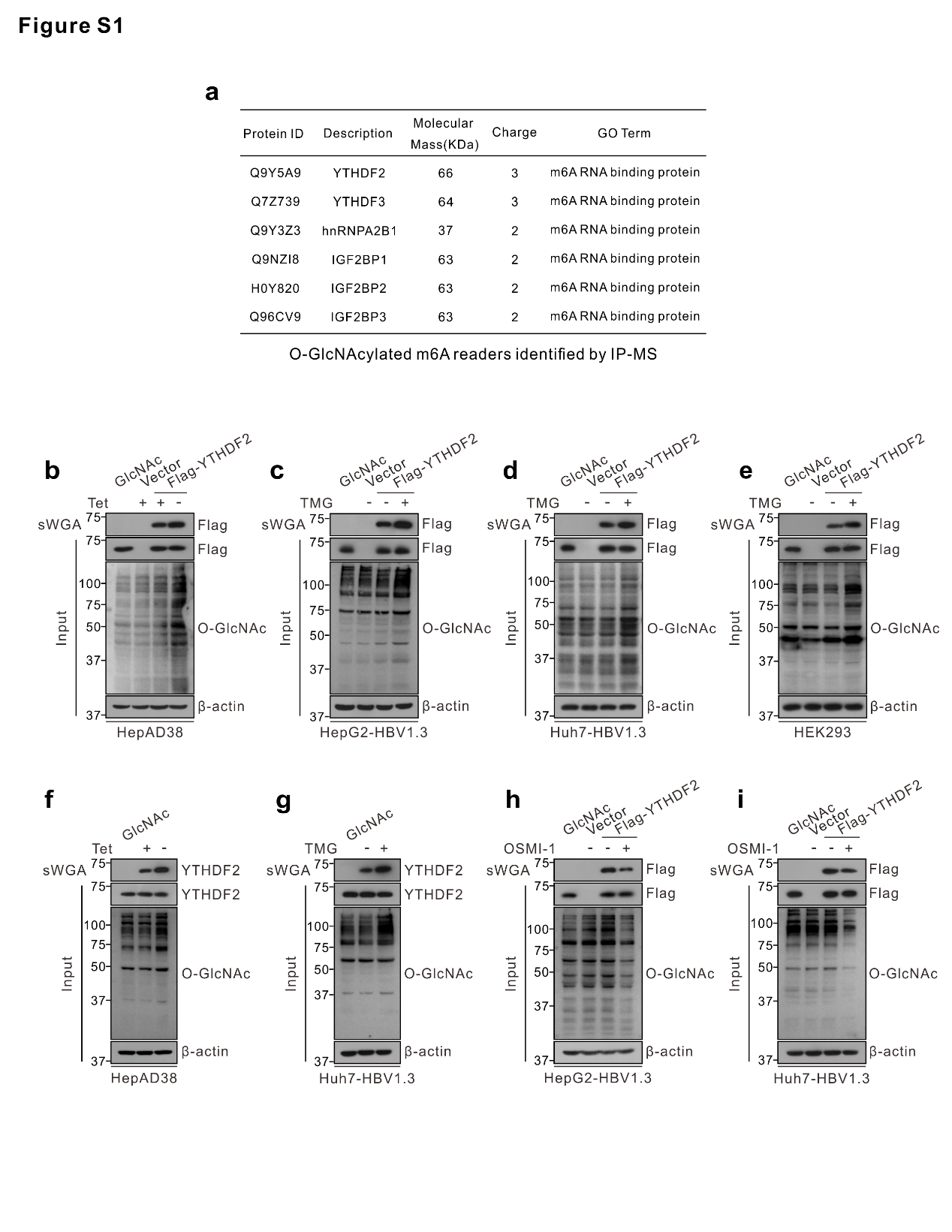


**Figure. S1 YTHDF2 O-GlcNAcylation is enhanced upon HBV infection. a** Gene ontology (GO) analysis of O-GlcNAcylated m^6^A readers identified using IP-MS in HepAD38 cells. **b**-**e** Exogenous succinylated wheat germ agglutinin (sWGA) pull-down assays were performed in HepAD38 cells without tet (**b**), AdHBV1.3-infected HepG2 cells (**c,** HepG2-HBV1.3), AdHBV1.3-infected Huh7 cells (**d,** Huh7-HBV1.3) and HEK293 cells (**e**) treated with 25 μΜ TMG. Cells were transfected with Flag-YTHDF2 or a vector control and western blotting was determined by anti-Flag. **f**, **g** Endogenous sWGA pull-down assays were performed in HepAD38 cells without tet (**f**) and Huh7-HBV1.3 cells (**g**) treated with 25 μΜ TMG for 12 h. Western blotting was determined by anti-YTHDF2. **h**, **i** Exogenous sWGA pull-down assays were performed in HepG2-HBV1.3 cells (**h**) and Huh7-HBV1.3 cells (**i**) treated with 20 μΜ OSMI-1 for 12 h. All the presented input was adjusted to a similar level for the following sWGA binding assay.


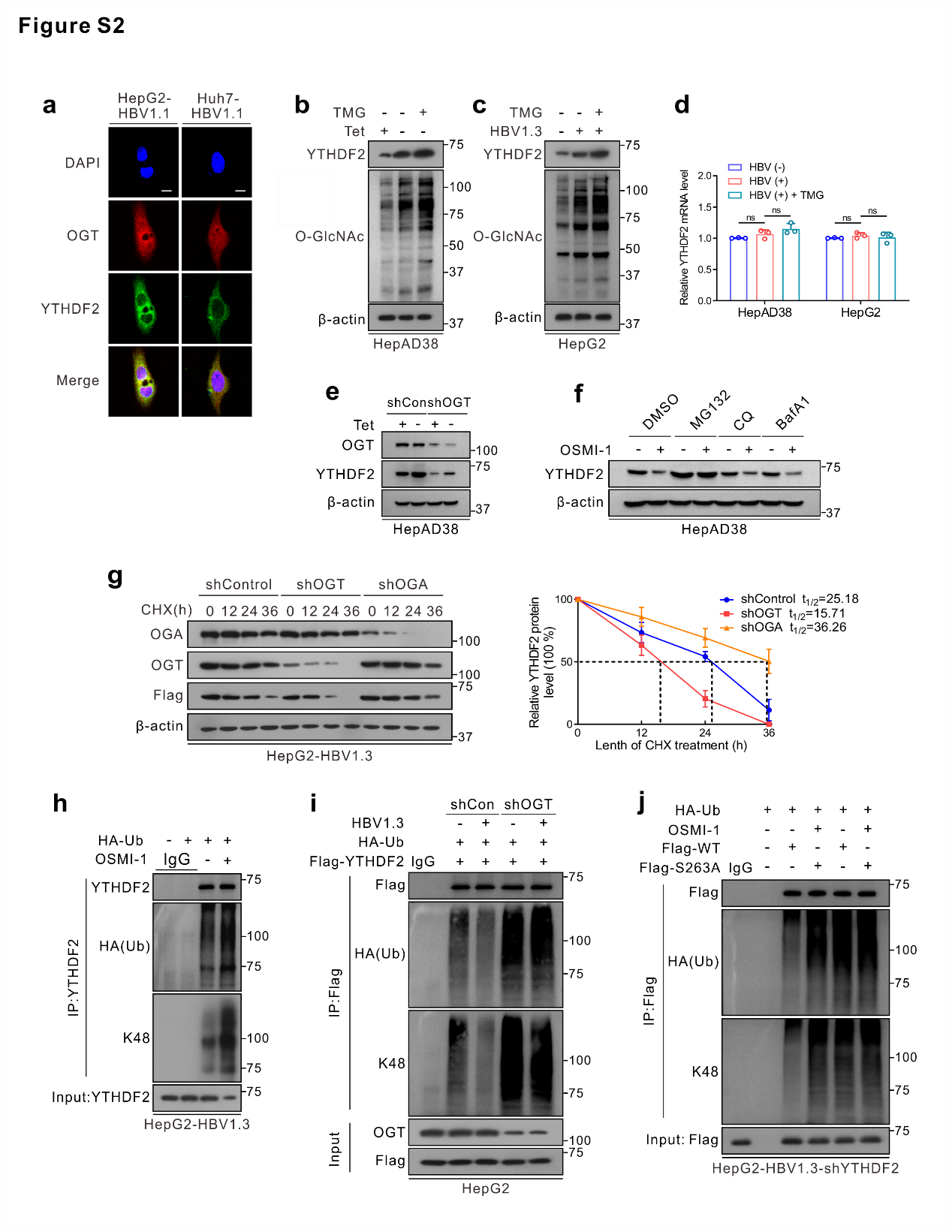


**Figure. S2 O-GlcNAcylation stabilizes YTHDF2 through suppression of its ubiquitination. a** Subcellular colocalization of YTHDF2 and OGT in HBV1.1-transfected HepG2 and Huh7 cells was determined by immunofluorescence staining (IF) (scale bars = 25 μm). **b**, **c** YTHDF2 protein expressions in HepAD38 cells (**b,** without tet) and HepG2 cells (**c,** infected with AdHBV1.3) treated with 25 μΜ TMG were determined by immunoblot analysis. **d** YTHDF2 mRNA levels in HepAD38 cells and HepG2 cells treated as described in **b**-**c** (n = 3, performed in triplicate). **e, f** YTHDF2 expressions detected with anti-YTHDF2. **e** HepAD38 cells were transfected with OGT shRNA lentiviral vector with or without tet. **f** HepAD38 cells were treated with 10 μM MG132, 50 μM chloroquine (CQ) or 100 nM bafilomycin A1 (BafA1) for 6 h before treatment with 20 μM OSMI-1. **g** Half-life and quantitative analysis of Flag-YTHDF2 in HepG2-HBV1.3 cells transfected with OGT, OGA, or Control shRNA lentiviral vectors (n = 3, performed in triplicate). The basal levels of Flag-YTHDF2 expression at 0 h were adjusted to a similar level for comparison. Data are expressed as the mean ± SD in **d** and **g** (One-way ANOVA). **h** YTHDF2 ubiquitination in HepG2-HBV1.3 cells in the presence of HA-tagged ubiquitin (HA-Ub). Cells were treated with 20 μM OSMI-1. After cell lysis, YTHDF2 was immunoprecipitated using anti-YTHDF2 antibody. **i, j** HepG2-HBV1.3 cells were co-transfected with HA-Ub and Flag-YTHDF2 (WT or S263A), followed by transfected with OGT shRNA lentiviral vector **(i)** or OSMI-1 treatment **(j).** YTHDF2 ubiquitination was determined by anti-Flag.


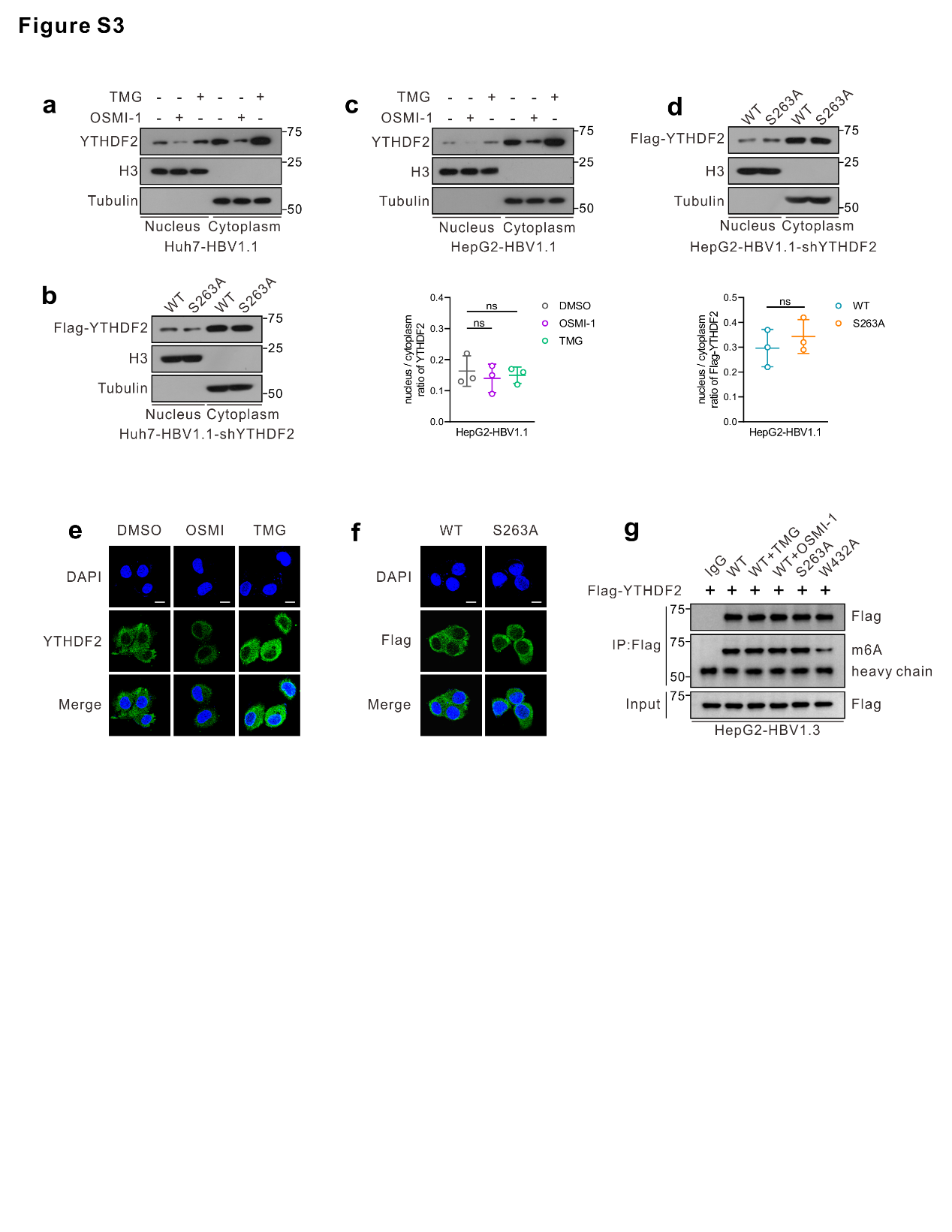


**Figure. S3 O-GlcNAcylation has minimal effect on subcellular localization and m^6^A binding affinity of YTHDF2. a, b** Representative images of subcellular localization of YTHDF2 in Huh7-HBV1.1 cells. **c-f** Subcellular localization of YTHDF2 in HepG2-HBV1.1 cells were determined by immunoblot analysis (**c, d**) or immunofluorescence staining (scale bars = 25 μm) (**e, f**). Nuclear and cytosolic fractions were immunoblotted with anti-YTHDF2 or anti-Flag, and the nucleus/cytoplasm ratio of YTHDF2 was quantified (n = 3, performed in triplicate) (**c, d**). **c, e** Huh7-HBV1.1 cells were treated with 25 μM TMG or 20 μM OSMI-1 for 24 h; **d, f** Huh7-HBV1.1 cells with YTHDF2 knockdown (shYTHDF2) were transfected with Flag-YTHDF2 (WT or S263A). **g** The abundance of m^6^A bound by YTHDF2 was detected by IP method. HepG2-HBV1.3 cells were transfected with Flag-YTHDF2 (WT, S263A or W432A); WT YTHDF2 were treated with 25 μM TMG or 20 μM OSMI-1, respectively. Cells were UV-crosslinked before harvest. Lysates were used for IP by anti-Flag antibody, and then immunoblotted with antti-m^6^A or anti-Flag antibodies.


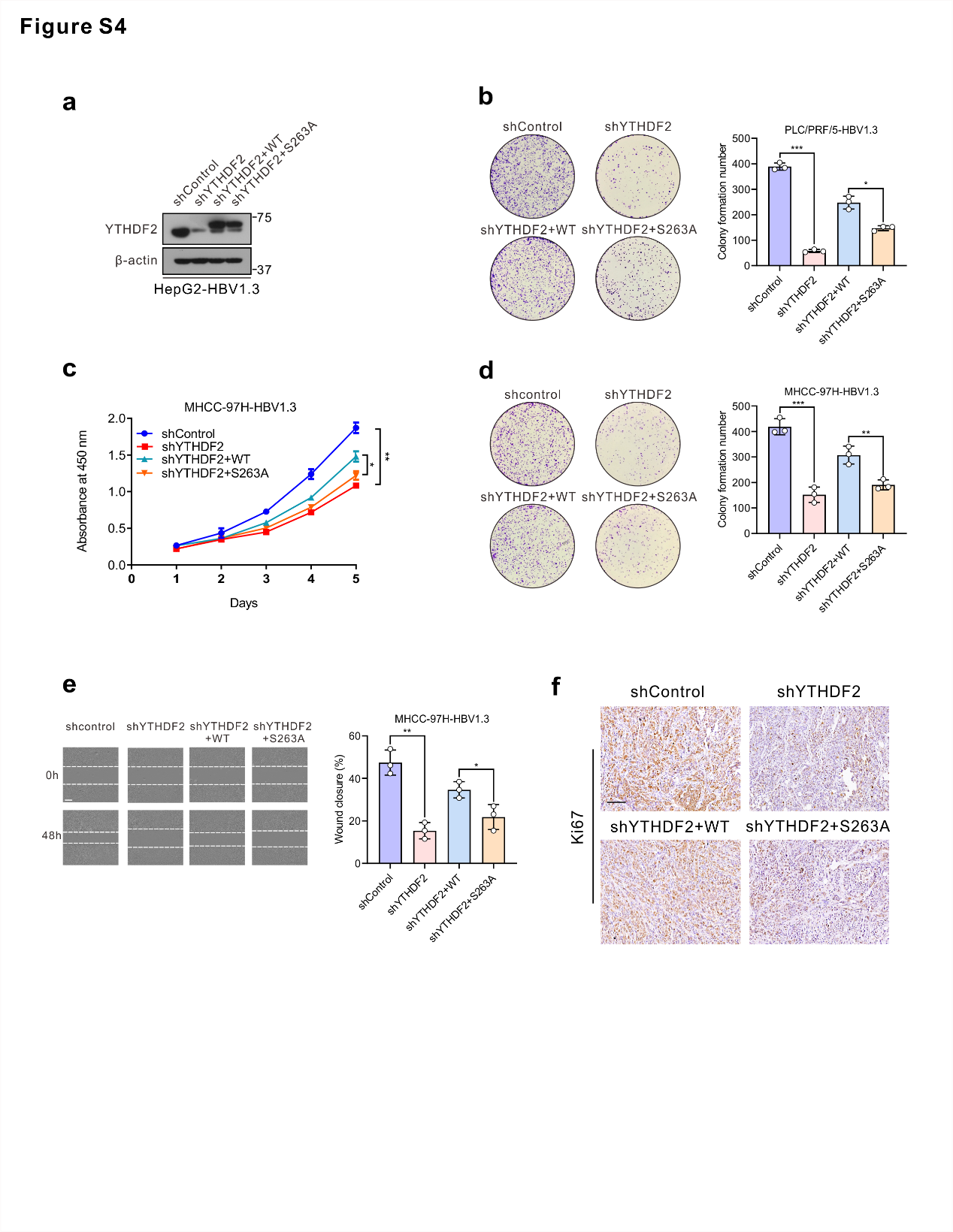


**Figure. S4 O-GlcNAcylation of YTHDF2 promotes hepatoma cell proliferation, invasion and migration *in vitro* and *in vivo*.** Cells were transfected with YTHDF2 shRNA lentiviral vector to knock down endogenous YTHDF2, and subsequently infected with adenoviruses expressing Flag-YTHDF2 (WT or S263A). All hepatoma cells were infected with AdHBV1.3. **a** YTHDF2 expression treated as described above in HepG2-HBV1.3 cells. **b-e** Colony formation capacity of PLC/PRF/5-HBV1.3 cells (**b**) and MHCC-97H-HBV1.3 cells (**d**) treated as indicated, n = 3. Cell proliferation ability (**c**) and migration capacity (**e,** bar = 200 μm) of MHCC-97H-HBV1.3 cells were detected by CCK8 and wound-healing assays treated as indicated, n = 3. Data are represented as mean ± SD. One-way ANOVA followed by Tukey’s test, **P* < 0.05, ***P* < 0.01, ****P* < 0.001. **f** Immunohistochemical (IHC) staining of Ki67 in subcutaneous implantation tumors, bar = 100 μm.


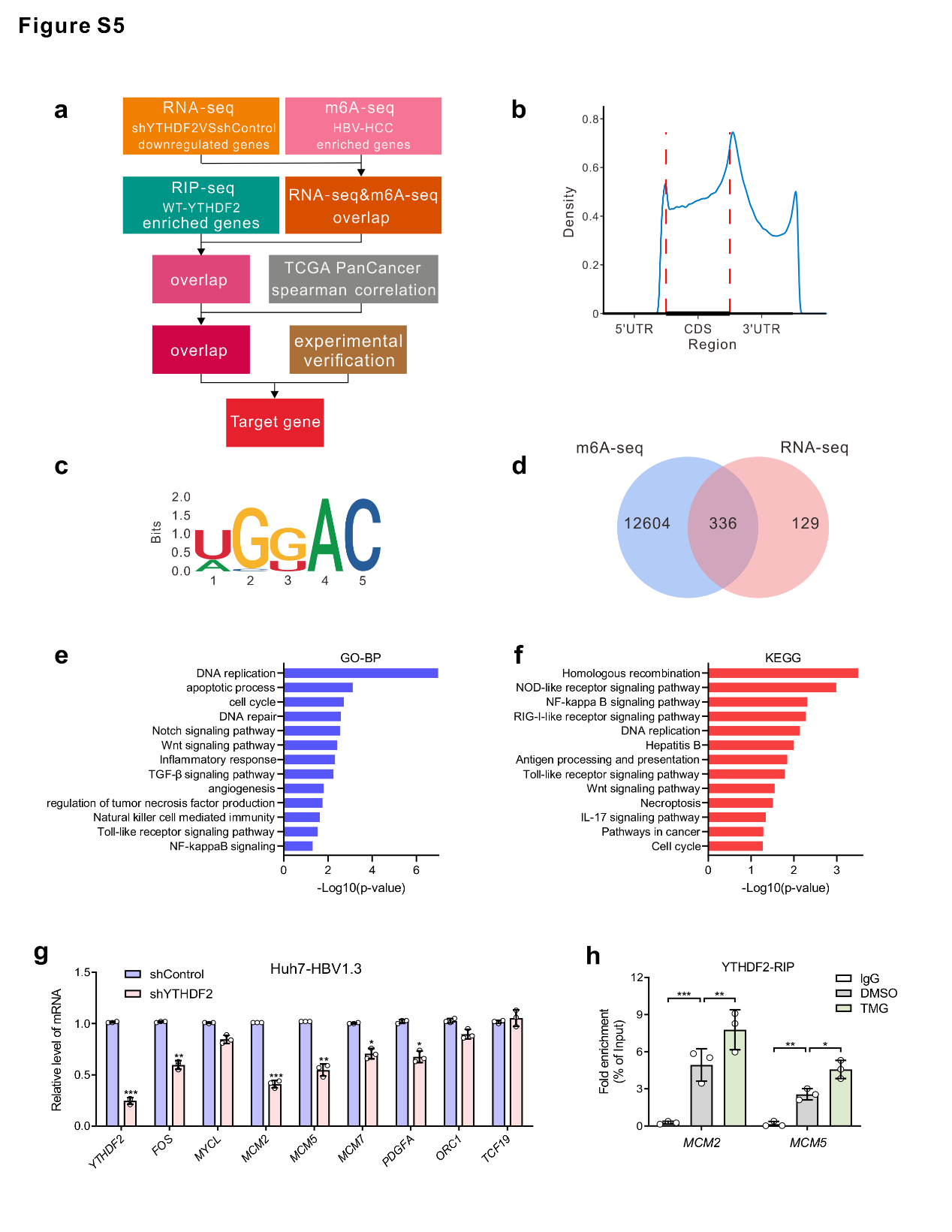


**Figure. S5 Identification of YTHDF2 targets by high-throughput RNA-seq, m^6^A-seq and RIP-seq. a** Schematic workflow for YTHDF2 downstream target identification. **b** Metagene profiles of m^6^A enrichment across the mRNA transcriptome in HBV-infected HepG2 cells. **c** m^6^A consensus sequence motif was identified in HBV-infected HepG2 cells. **d** Venn diagram illustrating overlapping targets of RNA-seq (downregulated upon YTHDF2 knockdown) and m^6^A-seq. **e**, **f** GO analysis of biological process (**e**) and KEGG enrichment analysis (**f**) of overlapping DEGs identified by RNA-seq and m^6^A-seq. **g** Relative mRNA levels of the initial screening genes in Huh7-HBV1.3 cells identified by RT-qPCR (n = 3, performed in triplicate). **h** YTHDF2-RIP-qPCR showing the association between *MCM2*/*MCM5* transcripts and YTHDF2 in Huh7-HBV1.3 cells (n = 3, performed in triplicate). For **g** and **h**, data are represented as mean ± SD, and analyzed by one-way ANOVA followed by Tukey’s test, **P* < 0.05, ***P* < 0.01, ****P* < 0.001.


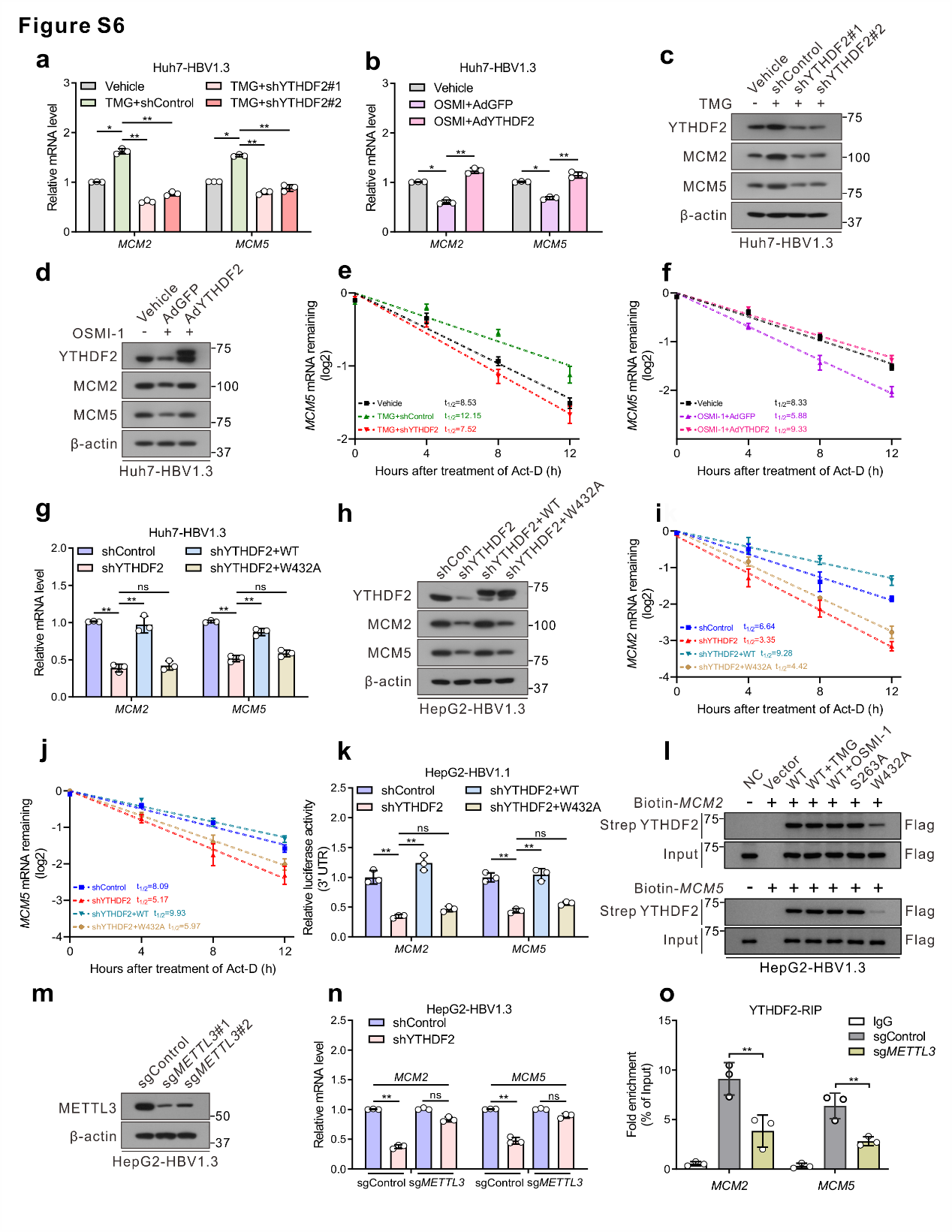


**Figure. S6 YTHDF2 stabilizes cell cycle-related gene *MCM2* and *MCM5* in an m^6^A dependent manner. a**-**d** Relative mRNA and protein levels of *MCM2* and *MCM5 (MCM2/5)*. Huh7-HBV1.3 cells were treated with 25 μM TMG, followed by transfected with control or two individual YTHDF2 shRNAs (**a**, **c**) or treated with 20 μM OSMI-1, and infected with AdGFP or AdYTHDF2 (**b**, **d**) (n = 3, performed in triplicate). **e**, **f** Lifetime of *MCM5* mRNA in HepG2 cells. Cells were treated with 25 μM TMG, followed by transfected with control or YTHDF2 shRNA (**e**) or treated with 20 μM OSMI-1, and infected with AdGFP or AdYTHDF2 (**f**). Transcription was inhibited by actinomycin D (5 μg/mL) (n = 3, performed in triplicate). **g**, **h** Cells were transfected with the YTHDF2 shRNA lentiviral vector, and subsequently transfected with Flag-tagged YTHDF2 (WT or W432A). **g** Relative mRNA levels of *MCM2/5* in Huh7-HBV1.3 cells, n = 3; **h** Protein levels of MCM2/5 in HepG2-HBV1.3 cells. **i**, **j** Lifetime of *MCM2/5* mRNA in HepG2-HBV1.3 cells (n = 3, performed in triplicate). Cells were transfected with YTHDF2 shRNA lentiviral, and subsequently transfected with Flag-tagged YTHDF2 (WT or W432A). **k** Relative luciferase activity of constructs containing 3’UTR of *MCM2* or *MCM5* in HepG2-HBV1.1 cells (n = 3, performed in triplicate). **l** Streptavidin agarose affinity pull-down assay using biotin-labeled *MCM2* or *MCM5*. HepG2-HBV1.3 cells were transfected with Flag-tagged YTHDF2 (WT, S263A or W432A), and treated with TMG or OSMI-1. After pull-down, YTHDF2 enrichments were detected with anti-Flag. **m** Protein level of METTL3 after transfected with sg*METTL3* or sgControl. **n** Relative mRNA levels of *MCM2/5* determined by RT-qPCR. HepG2-HBV1.3 cells were transfected with sg*METTL3* or sgControl lentiviral, followed by YTHDF2 shRNA lentiviral (n = 3, performed in triplicate). **o** YTHDF2-RIP-qPCR was performed in HepG2-HBV1.3 cells transfected with sg*METTL3* or sgControl lentiviral (n = 3, performed in triplicate). All experiments were performed in HBV-infected cells. Data are represented as mean ± SD. For **a**, **b**, **e**, **f**, **g**, **i**-**k**, and **n**-**o** data were analyzed by one-way ANOVA followed by Tukey’s test, **P* < 0.05, ***P* < 0.01, ****P* < 0.001.


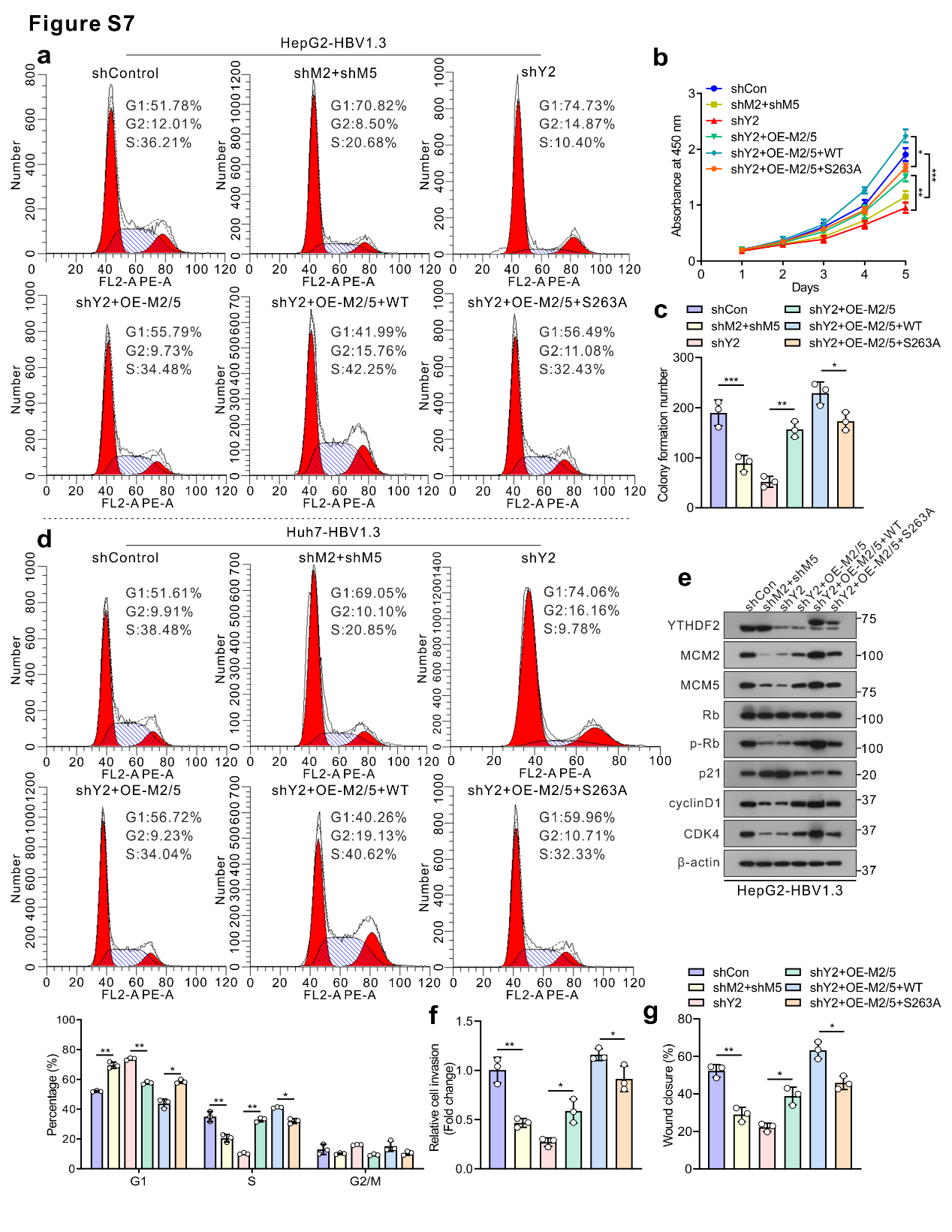


**Figure. S7 YTHDF2 stabilizes cell cycle-related gene *MCM2* and *MCM5* to promotes HCC proliferation. a** Representative images of cell cycle distribution in HepG2-HBV1.3 cells. **b-d** Huh7-HBV1.3 cells were transfected with YTHDF2 shRNA lentiviral vector (shY2) or MCM2 and MCM5 shRNA lentiviral vectors (shM2+shM5) to knockdown endogenous YTHDF2 or MCM2 and MCM5. Then shYTHDF2 groups were subsequently overexpressed with MCM2 and MCM5 (shY2+OE-M2/5), with (or without) overexpression of WT/ S263A YTHDF2. Cell proliferation (**b**), colony formation (**c**), and flow cytometric analysis (**d**) were determined as previously (n = 3, performed in triplicate). **e** HepG2-HBV1.3 cells were treated as described above, and cell cycle-related protein expressions were detected by western blot. **f, g** Cell invasion (**f**) and migration capacities (**g**) were determined as previously (n = 3, performed in triplicate). For **b-d, f** and **g**, data are represented as mean ± SD, and analyzed by one-way ANOVA followed by Tukey’s test, **P* < 0.05, ***P* < 0.01, ****P* < 0.001.


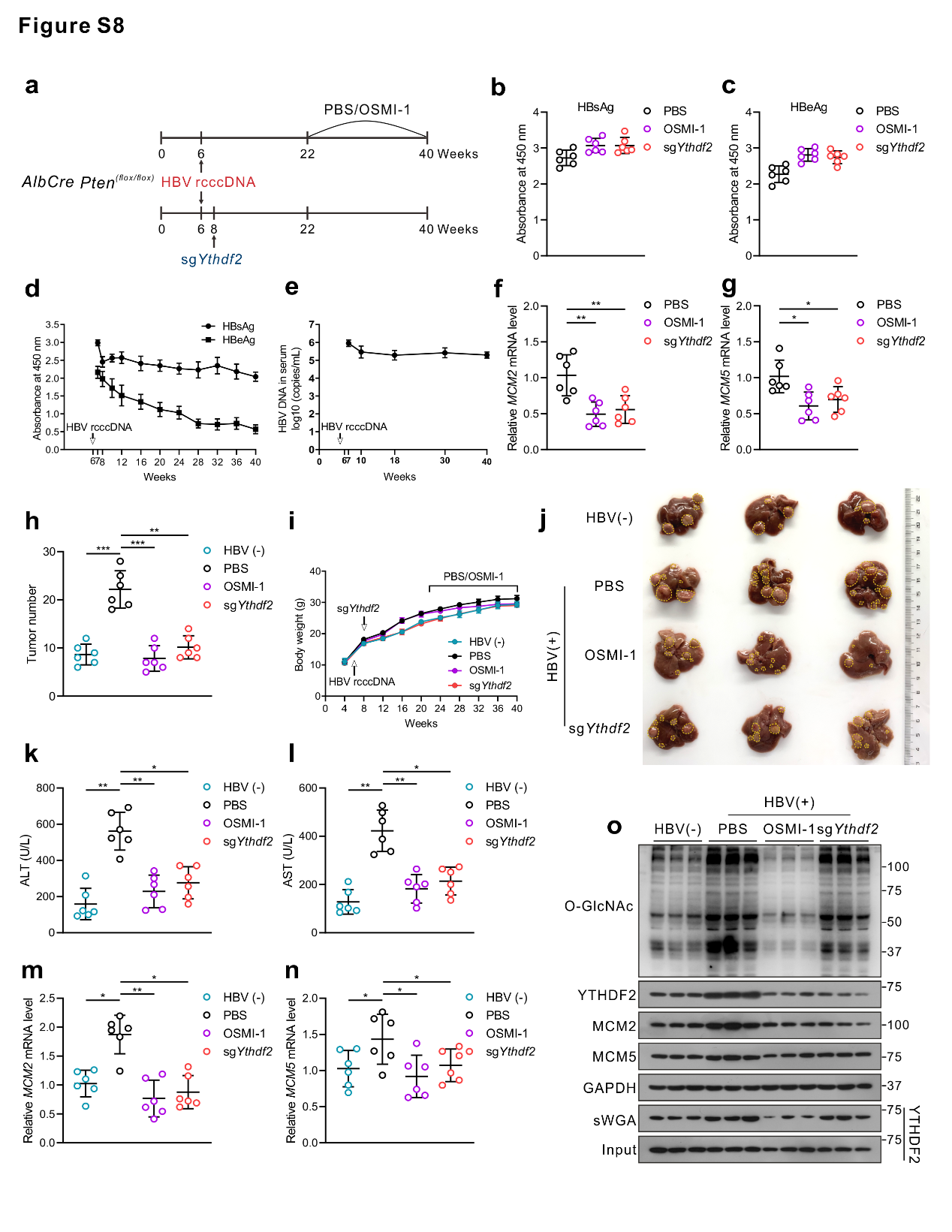


**Figure. S8 Targeting OGT-mediated YTHDF2 O-GlcNAcylation suppresses HBV-associated Hepatocarcinogenesis *in vivo*. a** Schematic showing the experimental procedures of HBV infection-induced *Alb-Cre* *Pten*^(flox/flox)^ mouse model. **b**, **c** HBsAg and HBeAg levels in the serum of HBV-Tg mice. **d**, **e** HBsAg, HBeAg and HBV DNA levels in serum of Alb-Cre mice were determined at indicated time. **f, g** mRNA levels of *MCM2* (**f**) and *MCM5* (**g**) in the liver tumors of HBV-Tg mice by RT-qPCR, n = 6/group. **h, i** Tumor nodule number (**h**) and body weight (**i**) of the Alb-Cre mice, n = 6/group. **j** Gross appearance of liver samples with tumors of Alb-cre mice. **k**, **l** ALT (**k**) and AST (**l**) levels in the mouse serum samples, n = 6/group. **m**, **n** mRNA levels of *MCM2* (**m**) and *MCM5* (**n**) in liver tumors of Alb-Cre mice by RT-qPCR, n = 6/group. **o** The indicated protein expressions and YTHDF2 O-GlcNAcylation levels in liver tumors of Alb-Cre mice by immunoblots analysis and sWGA pull-down assay. Data are represented as mean ± SD. For **f-h**, and **k**-**n**, data were analyzed by one-way ANOVA followed by Tukey’s test, **P* < 0.05, ***P* < 0.01, ****P* < 0.001.


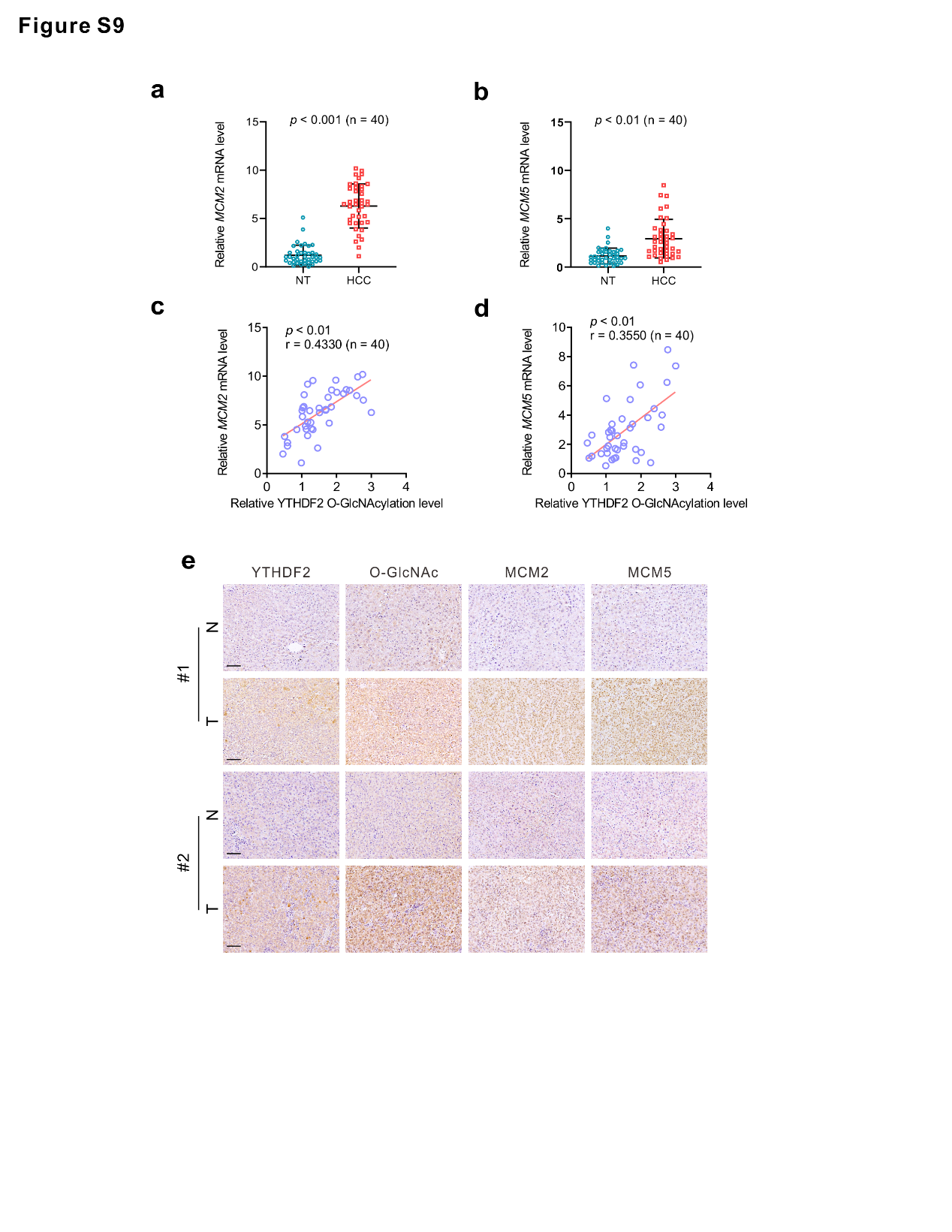


**Figure. S9 YTHDF2 O-GlcNAcylation is positively correlated with *MCM2* and *MCM5* to promote human HBV-related HCC progression. a**, **b** Relative mRNA levels of *MCM2* and *MCM5* in 40 paired clinical HBV-associated HCC tissue samples, *P* < 0.01 or *P* < 0.001. **c, d** Correlation analysis between YTHDF2 O-GlcNAcylation level and *MCM2* (**c**) or *MCM5* (**d**) mRNA levels in 40 paired clinical HBV-associated HCC tissue samples, *P* < 0.01. **e** IHC staining of the indicated proteins in clinical HBV-associated HCC tissues, bar = 100 μm.

**Table. S1** **Primers and oligos used in this study.**

| **Name** | **Sequence (5’**>**3’)** |
| --- | --- |
| **Oligos for shRNA** | |
| shOGT | Forward1:  TGCATGTTATTTGAAAGCAATTCAAGAGATTGCTTTCAAATAACATGCTTTTTTC  Reverse1:  TCGAGAAAAAAGCATGTTATTTGAAAGCAATCTCTTGAATTGCTTTCAAATAACATGCA  Forward2:  TGCCCTAAGTTTGAGTCCAATTCAAGAGATTGGACTCAAACTTAGGGCTTTTTTC  Reverse2:  TCGAGAAAAAAGCCCTAAGTTTGAGTCCAATCTCTTGAATTGGACTCAAACTTAGGGCA |
| shOGA | Forward1:  TGGACAATTCTTTATGACATTTCAAGAGAATGTCATAAAGAATTGTCCTTTTTTC Reverse1:  TCGAGAAAAAAGGACAATTCTTTATGACATTCTCTTGAAATGTCATAAAGAATTGTCCA  Forward2:  TGCCTTTGTACACTGCGGAATTCAAGAGTTCCGCAGTGTACAAAGGCTTTTTTC  Reverse2:  TCGAGAAAAAAGCCTTTGTACACTGCGGAATCTCTTGAATTCCGCAGTGTACAAAGGCA |
| shYTHDF2  (target YTHDF2-3'UTR) | Forward1:  TGCAACGGTTGCATCTGCATATTTCAAGAGAATATGCAGATGCAACCGTTGCTTTTTTC Reverse1:  TCGAGAAAAAAGCAACGGTTGCATCTGCATATTCTCTTGAAATATGCAGATGCAACCGTTGCA Forward2:  TGCAGCAACGGTTGCATCTGCATTCAAGAGATGCAGATGCAACCGTTGCTGCTTTTTTC Reverse2:  TCGAGAAAAAAGCAGCAACGGTTGCATCTGCATCTCTTGAATGCAGATGCAACCGTTGCTGCA |
| shMCM2 | Forward1:  TGCACAAGGTACGTGGTGATATTTCAAGAGAATATCACCACGTACCTTGTGCTTTTTTC Reverse1:  TCGAGAAAAAAGCACAAGGTACGTGGTGATATTCTCTTGAAATATCACCACGTACCTTGTGCA Forward2:  TGCATTGCTCCTTCCATCTATGTTCAAGAGACATAGATGGAAGGAGCAATGCTTTTTTC Reverse2:  TCGAGAAAAAAGCATTGCTCCTTCCATCTATGTCTCTTGAACATAGATGGAAGGAGCAATGCA |
| shMCM5 | Forward1:  TGCACGGGCTTCACCTTCAAATTTCAAGAGAATTTGAAGGTGAAGCCCGTGCTTTTTTC Reverse1:  TCGAGAAAAAAGCACGGGCTTCACCTTCAAATTCTCTTGAAATTTGAAGGTGAAGCCCGTGCA Forward2:  TGGATGAACTCAAGCGGCATTATTCAAGAGATAATGCCGCTTGAGTTCATCCTTTTTTC Reverse2:  TCGAGAAAAAAGGATGAACTCAAGCGGCATTATCTCTTGAATAATGCCGCTTGAGTTCATCCA |
| **Primers for recombinant DNA** | |
| pSEB-3Flag-YTHDF2 | Forward: ATAGCGGCCGCACCATGGGCATGTCGGCCAGCAGCCTCTTG Reverse: CGCGGATCCTTTCCCACGACCTTGACGTTC |
| pAdTrack-TO4- 3Flag- GFP-YTHDF2 | Forward: TGGGGTACCATGGGCTCGGCCAGCAGCCTCTT Reverse: CCGCTCGAGTTTCCCACGACCTTGACGTTC |
| pBudCE4.1-3HA-YTHDF2 | Forward: TGGGGTACCATGGGCTCGGCCAGCAGCCTCTT Reverse: GGAAGATCTCTTTTCCCACGACCTTGACGTTC |
| pBudCE4.1-3HA-FBW7 | Forward: TGGGGTACCATGGGCAATCAGGAACTGCTCTCTGTG Reverse: TGAAGATCTGACTTCATGTCCACATCAAAGTCC |
| pBuCE4.1-3HA-OGT | Forward: ATAGCGGCCGCACCATGGCGTCTTCCGTGGGC Reverse: CGCGGATCCTGCTGACTCAGTGACTTCAACAGG |
| pSEB-3Flag-OGT | Forward: ACGCGTCGACACCATGGCGTCTTCCGTGGGC Reverse: CGCGGATCCTGCTGACTCAGTGACTTCAACAGG |
| pAdTrack-TO4-GFP-MCM2 | Forward: TGGGGTACCATGGCGGAATCATCGG Reverse: TCCAAGCTTGAACTGCTGCAGGATCATT |
| pAdTrack-TO4-GFP-MCM5 | Forward: TGCGGATCCACCATGGGCTCGGGATTCGACGATCCT Reverse: TCCAAGCTTCTTGAGGCGGTAGAGAACCT |
| pGL3-Basic-3'UTR-MCM2 | Forward: TGGGGTACCTTTTATGACAGTGAGCTCTTC Reverse: GGAAGATCTTTTATATTTTATTCAACTTTATT |
| pGL3-Basic-3'UTR-MCM5 | Forward: TGGGGTACCAAATACCCGGAGCACGCCA Reverse: GGAAGATCTTTATGAGCCCCGCTAAACAGATG |
| **Primers for mutagenesis** | |
| pSEB-3Flag-S262A (YTHDF2 mutation  784T>G) | Forward: GGCATTGCAGGGGCAAGTCTTCCGC Reverse: GCGGAAGACTTGCCCCTGCAATGCC |
| pSEB-3Flag-S263A (YTHDF2 mutation  787-788AG>GC) | Forward: CATTGCAGGGTCAGCTCTTCCGCCACC Reverse: GGTGGCGGAAGAGCTGACCCTGCAATG |
| pSEB-3Flag-T524A (YTHDF2 mutation  1570A>G) | Forward: GAATAAACCAGTGGCCAACTCTAGGGACACTC Reverse: GTCCCTAGAGTTGGCCACTGGTTTATTCTCGT |
| pSEB-3Flag-W432A (YTHDF2 mutation 1294-1295 TG>GC) | Forward: TTAAGTATAATATTGCGTGCAGCACAGAGCATG Reverse: GCTCTGTGCTGCACGCAATATTATACTTAATGG |
| pBudCE4.1-3HA-ΔC (YTHDF2  1aa-400aa) | Forward: TGGGGTACCATGGGCTCGGCCAGCAGCCTCTT Reverse: GGAAGATCTCTGGGGTTATAGTTATTAATGGAC |
| pBudCE4.1-3HA-ΔN (YTHDF2 401aa-579aa) | Forward: TGGGGTACCATGAAAGATTTTGACTGGAATCTGA Reverse: GGAAGATCTCTTTTCCCACGACCTTGACGTTC |
| **Oligos for sgRNA** | |
| *Ythdf2*-Mouse-sgRNA-1 | Forward: CACCGAAGCTGCTTGGTCTACTGG Reverse: AAACCCAGTAGACCAAGCAGCTTC |
| *Ythdf2*-Mouse-sgRNA-2 | Forward: CACCGCAGTATATGCATTATTCTG Reverse: AAACCAGAATAATGCATATACTGC |
| *METTL3*-Human-sgRNA-1 | Forward: CACCGGTAATGACCTGACAGAGTT Reverse: AAACAACTCTGTCAGGTCATTACC |
| *METTL3*-Human-sgRNA-2 | Forward: CACCGTTGGAGACAATGCTGCCTC Reverse: AAACGAGGCAGCATTGTCTCCAAC |
| **Primers for quantitative RT-PCR** | |
| HBV core DNA (nt) 1770 to 2105 | Forward: CCTCTTCATCCTGCTGCT Reverse: AACTGAAAGCCAAACAGTG |
| YTHDF2-Human | Forward: CCAAAAGGTCAAGGAAACAAA Reverse: GGAGAAGCCAATGGAGGG |
| YTHDF2-Mouse | Forward: CCAAAAGGTCAAGGAAACAAA Reverse: GGAAAAGCCAATGGAGGG |
| FOS-Human | Forward: GGGGAGCCTTCAGAGAGAGT Reverse: TTCTTCCTGGGACGGTGAG |
| MYCL-Human | Forward: GGCTGGGGCAGGAACTAC Reverse: CGCTTGGGCTCTCGGAC |
| PDGFA-Human | Forward: TGTCAAGTGCCAGCCCTCC Reverse: CCGTGTCCTCTTCCCGATAAT |
| ORC1-Human | Forward: GAAGTCCCTGCCTGTAAACG Reverse: TGGGGCTAAAGGTATCACCC |
| TCF19-Human | Forward: TGCCATTACCATCCCACG Reverse: CAGGAGGCTCACTCTCATCATC |
| MCM7-Human | Forward: TAACTGTGCGTGGAATCGTCA Reverse: CCTGAGCGGTTGGTTTGG |
| MCM2-Human | Forward: GCCCGCTACCTTTCATTCC Reverse: TGATCTGACGAGCCTTATCCA |
| MCM2-Mouse | Forward: AGACTACCGTCCCATTCCG Reverse: TCTTCATCCTCCTCGCCAT |
| MCM5-Human | Forward: AGGCTCCCTGATGGACTTACT Reverse: CATGGCTTCGTGGATTGC |
| MCM5-Mouse | Forward: TGACGAGGAACTGGCTGACC Reverse: GCTGCGGCACTGAATGGA |
| β-actin-Human | Forward: AGGCCAACCGCGAGAAGATGACC Reverse: GAAGTCCAGGGCGACGTAGCAC |
| GAPDH-Human | Forward: CGACCACTTTGTCAAGCTCA Reverse: AGGGGTCTACATGGCAACTG |
| GAPDH-Mouse | Forward: CTCCCACTCTTCCACCTTCG Reverse: CCACCACCCTGTTGCTGTAG |
| **Oligos for RNA pull-down** | |
| Biotin-*MCM2* | 5’ to 3’: UGCGACUGAGGAGCCCAGAACG |
| Biotin-*MCM5* | 5’ to 3’: CGAGGCGUGGGCGAGUCCA |
